# Immunogenicity of the *Plasmodium falciparum* PfEMP1-VarO Adhesin: Induction of Surface-Reactive and Rosette-Disrupting Antibodies to VarO Infected Erythrocytes

**DOI:** 10.1371/journal.pone.0134292

**Published:** 2015-07-29

**Authors:** Micheline Guillotte, Alexandre Juillerat, Sébastien Igonet, Audrey Hessel, Stéphane Petres, Elodie Crublet, Cécile Le Scanf, Anita Lewit-Bentley, Graham A. Bentley, Inès Vigan-Womas, Odile Mercereau-Puijalon

**Affiliations:** 1 Institut Pasteur, Unité d'Immunologie Moléculaire des Parasites, Paris, France; 2 Centre National de la Recherche Scientifique, Unité de recherche associée 2581, Paris, France; 3 Institut Pasteur, Unité d'Immunologie Structurale, Paris, France; 4 Centre National de la Recherche Scientifique, Unité de recherche associée 2185, Paris, France; 5 Institut Pasteur, Plate-forme de Protéines recombinantes (PFPR), Paris, France; 6 Bordeaux Biothèques Santé, Groupe hospitalier Pellegrin, Centre Hospitalier Universitaire de Bordeaux - Bordeaux, France; Ehime University, JAPAN

## Abstract

Adhesion of *Plasmodium falciparum*-infected red blood cells (iRBC) to human erythrocytes (i.e. rosetting) is associated with severe malaria. Rosetting results from interactions between a subset of variant PfEMP1 (*Plasmodium falciparum* erythrocyte membrane protein 1) adhesins and specific erythrocyte receptors. Interfering with such interactions is considered a promising intervention against severe malaria. To evaluate the feasibility of a vaccine strategy targetting rosetting, we have used here the Palo Alto 89F5 VarO rosetting model. PfEMP1-VarO consists of five Duffy-Binding Like domains (DBL_1-5_) and one Cysteine-rich Interdomain Region (CIDR_1_). The binding domain has been mapped to DBL1 and the ABO blood group was identified as the erythrocyte receptor. Here, we study the immunogenicity of all six recombinant PfEMP1-VarO domains and the DBL1- CIDR1 Head domain in BALB/c and outbred OF1 mice. Five readouts of antibody responses are explored: ELISA titres on the recombinant antigen, VarO-iRBC immunoblot reactivity, VarO-iRBC surface-reactivity, capacity to disrupt VarO rosettes and the capacity to prevent VarO rosette formation. For three domains, we explore influence of the expression system on antigenicity and immunogenicity. We show that correctly folded PfEMP1 domains elicit high antibody titres and induce a homogeneous response in outbred and BALB/c mice after three injections. High levels of rosette-disrupting and rosette-preventing antibodies are induced by DBL1 and the Head domain. Reduced-alkylated or denatured proteins fail to induce surface-reacting and rosette-disrupting antibodies, indicating that surface epitopes are conformational. We also report limited cross-reactivity between some PfEMP1 VarO domains. These results highlight the high immunogenicity of the individual domains in outbred animals and provide a strong basis for a rational vaccination strategy targeting rosetting.

## Introduction

A hallmark of *Plasmodium falciparum* is the ability of its mature intracellular blood stages to cytoadhere to microvascular endothelial cells or circulating blood cells, causing vascular obstruction and local inflammation [1, 2]. The major parasite adhesin implicated in cytoadherence is PfEMP1 (*Plasmodium falciparum* erythrocyte membrane protein 1), a variant surface protein encoded by the approximately 60-member *var* gene family [3]. PfEMP1 adhesins contribute to pathogenicity by determining parasite binding to specific tissues or particular anatomical niches, and by enabling infected red blood cells (iRBCs) to evade host immunity [4]. Rosetting (the capacity of iRBC to cytoadhere to uninfected RBC) is consistently associated with severe malaria in African children [5–8] and a high parasite burden in a non-human primate experimental model [9]. Rosetting is due to expression of a subset of *var* genes from the so called UpsA group [10–12] that code for adhesins capable of binding to a variety of receptors on the RBC surface [10, 13–16]. A reasonable strategy to prevent malaria pathology is thus to target rosette-forming PfEMP1 adhesins by either vaccination or soluble inhibitors.

Immunogenicity studies are an essential step towards vaccination in order to identify antigen constructs and production systems as well as immunisation regimens that reproducibly elicit a protective response in outbred animals. In the case of PfEMP1 adhesins, specific challenges stem from the modular structure of the extra-cellular region, which consists of an array of Duffy-Binding Like (DBL) and Cysteine-rich Inter-Domain Region (CIDR) domains. The DBL and CIDR sequences and domain arrangements vary between paralogs within the PfEMP1 repertoire and between repertoires [3]. They can be assigned to a limited number of classes based on specific sequence signatures [4, 17]. Each module contains approximately 300–350 amino acids and includes numerous cysteine residues engaged in disulfide bonds [18–20].

Most immunogenicity studies on PfEMP1 concern PfEMP1-Var2CSA, the variant adhesin that promotes sequestration in the placenta of pregnant women [21]. Different expression systems have been explored to produce single domain or multidomain constructs [22–27]. Apart from immunisation using DNA constructs [28, 29], responses elicited by recombinant proteins were variable, with a wide range of titres, variable proportions of immunised animals producing cytoadherence-inhibiting antibodies [22–27, 30–36] and discordant results in different species of laboratory animals [22, 33, 34]. Systematic immunogenicity studies are scarce for rosette-forming PfEMP1 adhesins. Recently, immunisation data in groups of three rats showed substantial variability of ELISA titres but consistent production of surface reacting antibodies [30]. Ghumra et al. [14] immunised groups of two rabbits to produce antibodies against each individual domain of PfEMP1 IT4var9/R29 variant protein, which promotes rosetting [37] and adhesion to human brain vascular endothelium cells in vitro [38], and reported inter-animal variability with regard to iRBC surface reactivity.

Here, we use the model of the rosette-forming PfEMP1-VarO adhesin [9, 16, 39] to explore the immunogenicity of the individual domains in outbred and inbred mice and for the adhesion domain, in the rabbit as well. The PfEMP1-VarO extracellular domain has 5 DBL domains (DBL_1-5_) and one CIDR domain (CIDR_1_). We compare the immunogenicity of some recombinant PfEMP1 domains produced in different expression systems (baculovirus/insect cells, *Pichia pastoris* and *Escherichia coli*). We analyse the dynamics of antibody production in individual BALB/c and outbred mice immunised using a uniform regimen. Responses were monitored using several readout assays: ELISA on recombinant domains, immunoblotting of reduced and non-reduced PaloAlto 89F5 VarO protein extracts, the capacity to react with the native, surface-exposed PfEMP1-VarO as assessed by surface immunofluorescence and the capacity to prevent formation or disrupt PaloAlto 89F5 VarO rosettes. We studied the influence of protein folding on the production of surface-reacting antibodies and explored inter-domain cross-reactivity. These results underline the high immunogenicity of each individual domain and provide a strong basis for a rational vaccination strategy.

## Materials and Methods

### Palo Alto Var O parasites

The 89F5 PaloAlto VarO clonal parasite line was cultivated as described [39]. To maintain a monovariant line expressing the *varO* gene, rosetting parasites were enriched once a week on ice-cold Ficoll and rosetting rate was kept > 90% by panning on a specific monoclonal antibody as described [39, 40]. Procedures and reagents for culture, rosette purification and rosette formation assay have been published in detail [41].

### Production of recombinant VarO domains

The list of domains produced and their expression systems is shown in Table 1. The wild type eDBL0 sequence (DBL1α_1_ devoid of NTS) was cloned in pET-22b with an in-frame C-terminal His tag and expressed in BL21(DE3)pLysS *E*. *coli* competent cells (Invitrogen, Life technologies). Culture conditions were as recommended by the supplier (Invitrogen, https://tools.lifetechnologies.com/content/sfs/manuals/oneshotbl21_man.pdf), and optimised as follows: culture was performed at 37°C in the presence of 50 μg.mL^-1^ ampicillin and 34 μg.mL^-1^ chloramphenicol. Induction with IPTG was performed at 30°C for 1 hr. Extraction of the protein was performed using BugBuster (Novagen) with Benzonase (25 U.mL^-1^ of solution), 1x protease inhibitor cocktail (Roche Diagnostics). The lysate was centrifuged 20 min at 16,000g at 20°C. The pellet containing the insoluble protein (inclusion bodies) was washed with 1 M urea, 10 mM DTT for 15 min at RT, recovered by centrifugation at 16,000 g for 20 min at 20°C, and resuspended in 6 M urea.

**Table 1:** List of recombinant PfEMP1-VarO domains studied, sequence features and expression system used.

VarO domain (Type)	Name used in this work	PfEMP1-VarO amino acid residues	Sequence features (No glycosylation sites mutated S/T to A)	Expression system	Reference
DBL1 (α6)	eDBL0	96–402	Native	*E*. *coli*/pET22b	This work
NTS-DBL1 (α1.6)	bDBL1	1–487	Recodoned (7)	Baculovirus/insect cells	[39]
	pDBL1	1–487	Recodoned (7)	*P*. *pastoris*/pPICzaA	[42]
	eDBL1	1–487	Recodoned (7)	*E*. *coli*/pMAL-c2X	[42]
Head (α1-γ6)	eHead	2–716	Recodoned (8)	*E*. *coli*/pMAL-c2X	[16]
CIDR (γ6)	bCIDR	399–835	Recodoned (1)	Baculovirus/insect cells	This work
	pCIDR	508–787	Recodoned (1)	*P*. *pastoris*/pPICzaA	This work
DBL2 (β7)	bDBL2	821–1242	Recodoned (2)	Baculovirus/insect cells	This work
	eDBL2	821–1242	Recodoned (2)	*E*. *coli*/pET22b	This work
DBL3 (γ14)	eDBL3	1220–1578	Recodoned (3)	*E*. *col*i/pET22b	[16]
DBL4 (ε5)	eDBL4	1608–2014	Recodoned (3)	*E*. *coli*/pMAL-c2X	[16]
DBL5 (ε4)	eDBL5	2025–2321	Recodoned (7)	*E*. *coli*/pMAL-c2X	[16]

Cloning of most recodoned individual domains and Head domain has been described previously [16, 19, 39, 42]. Recombinant baculovirus products were obtained using the Bac-N-Blue transfection and expression system (Invitrogen, Life technologies) according to the manufacturer's protocol. Viral stocks were produced in SF9 insect cells, titrated and stored at 4°C. The recombinant viral clones were confirmed by PCR and sequencing. SF9 cells (3.10^9^ cells) were infected with recombinant baculovirus for 3 to 5 days and the medium, containing the secreted recombinant proteins was then harvested by centrifugation for 25 min at 2500 rpm. Secreted proteins were first concentrated 10x by tangential flow filtration on a 10 kDa VIVAFLOW membrane (Sartorius, Stedim). Soluble proteins were subsequently dialyzed against phosphate buffer saline solution (DPBS, pH 7.5, Gibco) containing 500 mM NaCl. Purification steps were performed at 4°C, to minimize protein degradation. After incubation with Cobalt-charged resin (TALON metal affinity resin, Clontech), the His-tagged proteins were eluted with an imidazole gradient (0–200 mM). Protein preparations were analysed by SDS-PAGE and Western blot. The sequence of each recombinant protein was verified by N-terminal sequencing and mass spectrometry analysis.

The coding CIDR1γ sequence with a C-terminal His6 coding sequence was cloned into pPICZaA (Invitrogen, Life technologies) and expressed in *Pichia Pastoris* GS115 (Invitrogen, Life technologies). Cultures were run for 60 hr at 30°C with continuous shaking as recommended by the manufacturer and 1% methanol was added every 24 hr for induction. The soluble protein was purified by metal affinity as above (TALON metal affinity resin, Clontech), followed by exclusion chromatography (S200 16/60 or S75 16/60, Amersham).

Recombinant proteins were analysed by SDS-PAGE polyacrylamide gel and stained with Bio-safe Coomassie (Bio-rad). Purity of each recombinant protein was verified by N-terminal sequencing and mass spectrometry analysis. Recombinant proteins were stored in aliquots at -80°C and thawed before use. This step caused internal cleavage in some proteins.

### Immunisation with recombinant proteins

**Fig 1:**
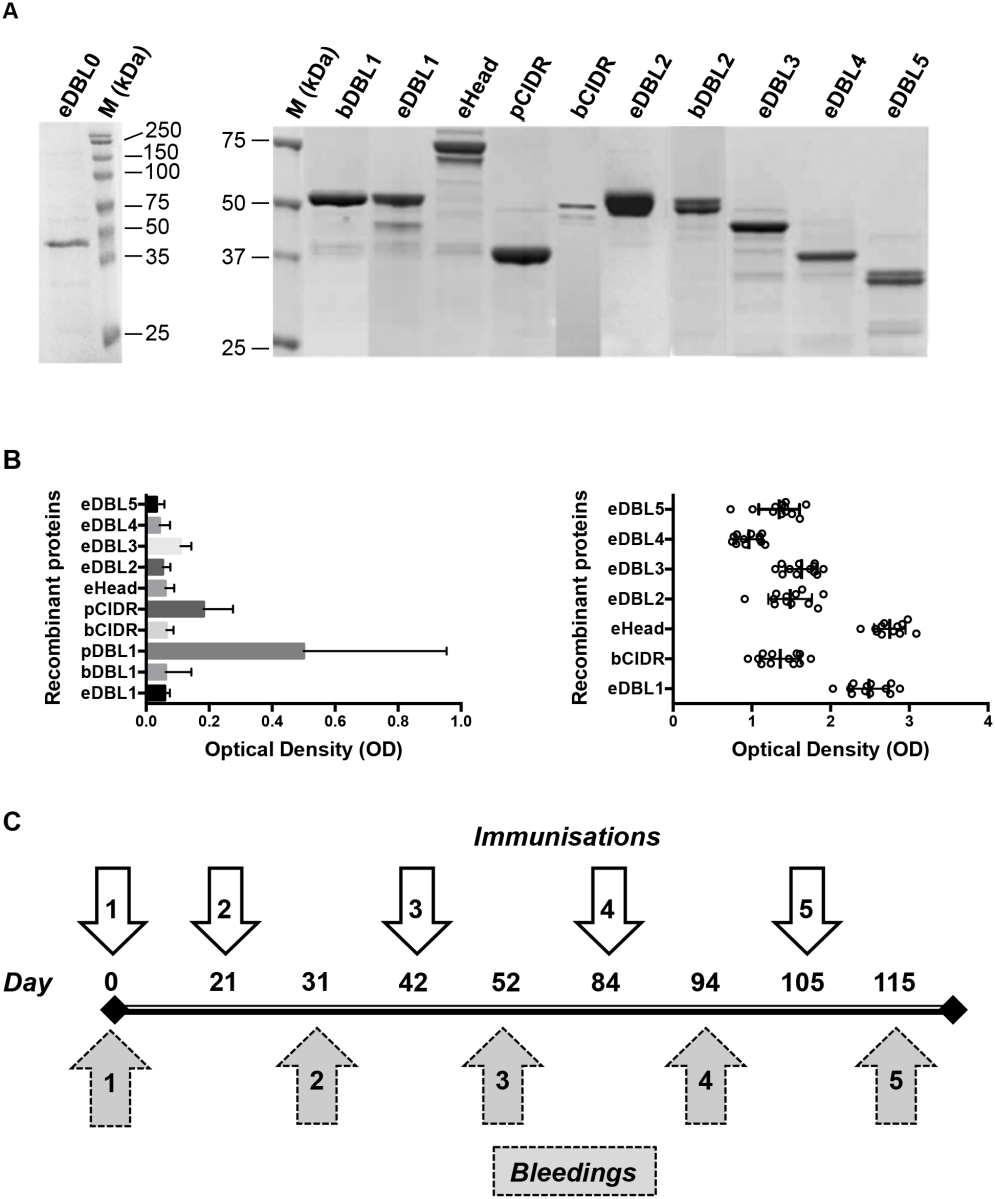
PfEMP1-VarO-derived antigens and immunisation schedule. **(A)** Coomassie blue staining of SDS-PAGE gels of the recombinant antigens used for immunisation and ELISA. The purity of the recombinant domains was assessed by electrophoresis of reduced proteins separated on 10% SDS PAGE gels. Antigens (1 μg per lane) were loaded as indicated. The molecular mass markers (M) are indicated. **(B)** ELISA assays of the recombinant proteins with human sera collected from 54 persons living in France and never exposed to malaria parasites (non-immune) (left panel) or individual sera from 12 Senegalese adults living in a malaria hyper-endemic setting (right panel). For details of ELISA see Materials and Methods. Please note that reactivity of individual human sera with bDBL1 is not included as it has been reported previously [39]. **(C)** Immunisation regimen and bleeding schedule used for all antigens.

Immunisations of mice were done with native or reduced recombinant proteins using a similar regimen for all antigens (Fig 1). Unless stated otherwise, immunisation with native, soluble recombinant protein was done in groups of 5–7 inbred (BALB/c) or outbred (OF1) female mice (6–8 week old, Charles River, France), which were injected subcutaneously at 4-week intervals, with 10 μg recombinant antigens For immunisation with a reduced antigen, the protein was first reduced with 20 mM DTT for 2h at 37°C and alkylated with 0.06 M iodoacetamide for 30 min at RT. For immunisation with eDBL0 solubilised in urea, 5 inbred (BALB/c) and 5 outbred (OF1) female mice were immunised 3 times at 3-week intervals subcutaneously for the first injection and intra-muscularly for the second and third one. All antigens were mixed with Freund’s adjuvant (complete for the first immunisation and incomplete for subsequent immunisations). Sera were collected before the first immunisation (D0) and 8–10 days after the second, third or fourth injection and stored at –20°C until use.

Two rabbits were custom immunised with 100 μg baculovirus-produced rNTS-DBL1α (RD Biotech, France). Immunoglobulins were purified by RD Biotech.

### Ethics statement

Human blood for parasite culture was purchased from the Etablissement Français du Sang. Supply and handling of human red cells followed the guidelines of the agreement between Institut Pasteur and the Etablissement Français du Sang and complied with the regulation of blood donation in France. The IMP Unit was issued an “Habilitation à manipuler du sang humain” (HS2003-3255, on 10^th^ October 2003; renewed 24^th^ February 2011; ref ND/LK/CC-11.68) under these conditions.

The study was carried out in strict accordance with the recommendations in the Guide for the Care and Use of Laboratory Animals of the Institut Pasteur (http://webcampus.pasteur.fr/jcms/c_283578/prodecures-approuvees-par-le-comite-d-ethique) and complied with the European Union guidelines for the handling of laboratory animals (http://ec.europa.eu/environment/chemicals/lab_animals/home_en.htm). The procedures were approved by the Institut Pasteur animal care and use committee. Animal care and handling was approved by the Ministère de l’Agriculture et de la Pêche (Ref 107503056792, issued to OMP) and the protocols and procedures approved by the Direction Départementale des Services Vétérinaires du Préfet de Police de Paris (clearance number C75–273 issued to OMP). All animal experiments were planned and executed in order to minimize animal suffering.

### ELISA assays

ELISA plates (Nunc maxisorp 439454, France) were coated with 100 μL/well of a solution of 0.2 μg.mL^-1^ recombinant protein in DPBS, overnight at 4°C. For reduced and alkylated antigens, proteins were reduced immediately after coating using 40 mM DTT (DL-Dithiothreitol, Sigma) in 200 mM Tris HCl, pH 8.8, 5.5 mM EDTA for 2 hr at RT followed by alkylation with 15 μL 0.4 M iodoacetamide for 20 min at RT. Non-specific absorption was blocked with 200 μL blocking buffer (DPBS, 5% w/v non-fat milk) for 1 hr at 37°C, 100 μL serial dilutions of sera (in DPBS, 2.5% non-fat milk, 0.05% Tween-20) were incubated 1 hr at 37°C. Plates were then washed three times with washing buffer (DPBS, 0.05% Tween-20) and incubated for 1 hr at 37°C with horseradish peroxidase-conjugated goat anti-mouse IgG (H+L) (Promega, France) diluted at 1/3,000 or anti-rabbit IgG (Promega, France) diluted 1/3,000 in washing buffer. Plates were washed three times before incubation for 5 min at RT with 100 μL TMB/H_2_O_2_ substrate (KPL). The enzymatic reaction was stopped by addition of 100 μL 1M H_3_PO_4_. Absorbance was measured at 450–655 nm. Each serum dilution was tested in duplicate.

### Immunoblots of reduced and non-reduced VarO parasite extracts

VarO rosettes selected on ice-cold Ficoll were first enriched on magnetic columns (Miltenyi Biotec) to obtain a preparation containing 95% iRBCs at the mature stage [41]. Immunoblots were prepared as described [43] with minor modifications. Briefly, proteins were fractionated on 4–12% SDS-PAGE gradient and transferred to a nitrocellulose membrane (Amersham). The membranes were blocked with DPBS-milk (DPBS, 5% non-fat milk), 1 hr at RT and probed with diluted sera in DPBS-milk-Tween (PBS, 2.5% milk, 0.05% Tween 20). After 3 washes in DPBS-Tween (PBS, 0.1% Tween), the blot was incubated with anti-species IgG (H+L) conjugated to alkaline phophatase (Promega, France, dilution 1/10,000) in DPBS-Tween for 1 hr at RT, washed and binding revealed as recommended by the supplier.

### Surface Immuno-fluorescence assay

Antibodies (IgG) to PfEMP1-VarO protein displayed on iRBC surface were monitored by indirect immuno-fluorescence using flow cytometry or fluorescence microscopy. After rosette enrichment, an aliquot of 89F5 VarO rosetting culture (>85% rosetting rate) was resuspended at 2.5% hematocrit in DPBS/FCS medium (DPBS, 2% foetal calf serum). Parasite suspension (20–40 μL) was incubated 30 min at 37°C with plasma or serum diluted 1/20. After 2 washes with DPBS/FCS, the cell pellet was resuspended in 100 μL DPBS/FCS medium containing goat anti human IgG (H+L) Alexa Fluor 488 conjugate (Molecular probes, A-11013, diluted 1/1,000) and Hoechst 33342 (Molecular probes, H-3570, diluted 1/1,000). After an incubation of 30 min at 37°C, parasite pellets were washed twice with DPBS/FCS medium and resuspended in 200 μL formaldehyde 0.37% (Sigma F-8775). Immuno-fluorescence staining was analysed as described [33].

### Rosette inhibition and rosette disruption assays

Rosette inhibition and rosette disruption assays were performed as described in detail elsewhere [41, 44]. Briefly, for rosette inhibition rosette-enriched Palo Alto VarO monovariant cultures (20 μL, 5% parasitaemia) were first disrupted with 10 μg.mL^-1^ Dextran sulphate 500, 000, which was extensively washed out using RPMI medium supplemented with 1% Human AB^+^ serum. After centrifugation, the cell pellet was resuspended in complete culture medium in the presence of either pre-immune or immune mouse serum, incubated for 30 min at 37°C and the rosetting rate was assessed [41]. For rosette disruption, rosette-enriched Palo Alto VarO monovariant cultures (20 μL, 5% parasitaemia) in complete RPMI culture medium were incubated with pre-immune or immune sera (final dilution 1/20 or lower). The rosetting rate was evaluated by light microscopy after iRBC nuclei staining with Hoechst dye. The rosetting rate was calculated by determining the percentage of rosette-forming iRBC present in the mature parasite population, the 100% rosetting rate being without added mouse serum. Each serum was tested in duplicate and two or three independent experiments were performed.

## Results

### Expression constructs: solubility and antigenicity

The constructs and expression systems used are listed in Table 1. To maximize the chance of producing these cysteine-rich domains as correctly folded recombinant proteins, three expression systems were tested for some domains: i) baculovirus/insect cells (Sf9 or HiFive cells) [39], ii) *Pichia pastoris* [42] and iii) *Escherichia coli* [16, 42]. All expression products were obtained as soluble proteins except for eDBL0, which was produced mostly as an insoluble protein in *E*. *coli* that was solubilised in urea. The proteins used for immunisation and ELISA assays were pure, as judged by absence of major contaminants in Coomassie blue-stained SDS-PAGE gels (Fig 1A) as well as N-terminal sequencing (data not shown) [16, 42].

Antigenicity of the constructs (except eDBL0 which was insoluble in the absence of urea) was assessed using immune sera from humans living in endemic areas and control sera from non-exposed adults living in France (Fig 1B, left panel). All constructs were readily recognised by immune sera (Fig 1B, right panel shows details for some constructs). The *Pichia pastoris* NTS-DBL1α (pDBL1) construct strongly reacted with non immune sera, but this was not observed with the baculovirus (bDBL1) or the *E*. *coli* (eDBL1) constructs (Fig 1B, left panel). However, we established that the elevated reactivity of pDBL1 with non immune sera was due to O-glycoslyation by *Pichia pastoris* (data not shown) and the construct was therefore not studied further.

The immunisation regimen used for all proteins is shown Fig 1C. For a given antigen, inbred (BALB/c) or outbred animals (OF1) were immunised with the same adjuvanted preparation (see Materials and Methods section). Antibody responses were assessed for each individual animal.

### Number of doses required to elicit a homogeneous antibody response in all animals

**Fig 2:**
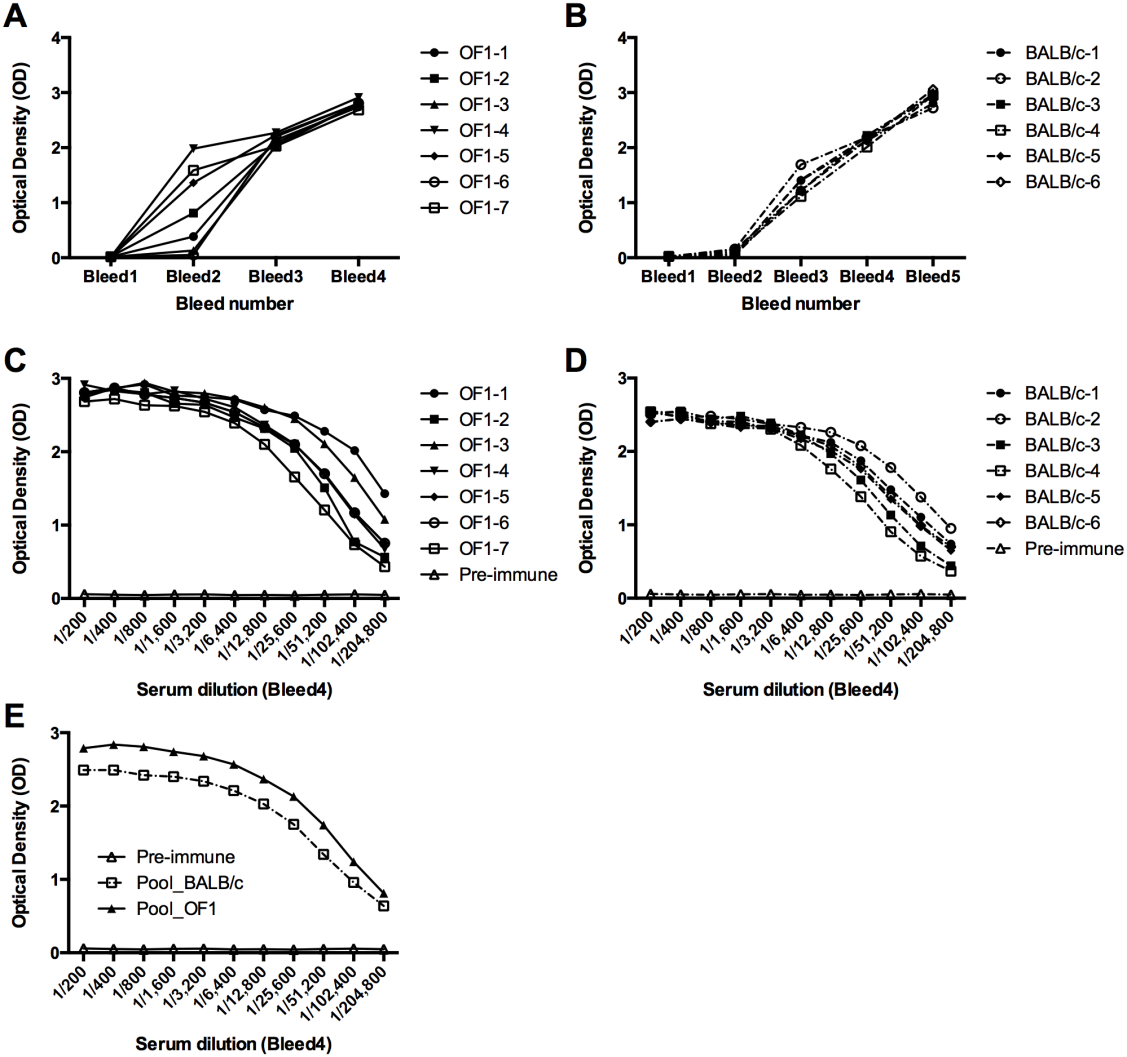
Homogeneity of the antibody response to bDBL2 after three immunising doses. ELISA analysis of the antibody response of inbred and outbred mice with increasing number of immunisations with PfEMP1-VarO bDBL2 domain. Sera collected from seven individual outbred mice (OF1-1 to -7) **(A, C)** or six BALB/c mice (BALB/c-1 to -6) **(B, D)** were assayed by ELISA on the cognate bDBL2 antigen. (**A, B)**: individual sera from successive bleedings assayed at a (dilution 1/200); (**C, D**): titration curves of individual sera from bleed 4 (same numbering of animals for A,C and B,D). **(E)** Comparative titration curves of the pools prepared by mixing equal volumes of individual sera from bleed 4 of OF1 or BALB/c mice.

We first analysed the number of doses needed to achieve a homogeneous ELISA response in OF1 and BALB/c mice using the baculovirus-expressed DBL2βC2 (bDBL2) and NTS-DBL1α_1_ (bDBL1) domains. Three injections were necessary to elicit a response to bDBL2 in all animals. After two injections, responses were heterogeneous in OF1 and minimal in BALB/c mice (Fig 2A and 2B, respectively); they substantially increased and were quite homogeneous after the 3rd injection in both OF1 and BALB/c mice. The high response was sustained after the 4th and 5th dose. All outbred and inbred animals produced good titres of anti-bDBL2 antibodies (Fig 2C and 2D), although signal intensity was usually higher in OF1 than in BALB/c mice (Fig 2E).

**Fig 3:**
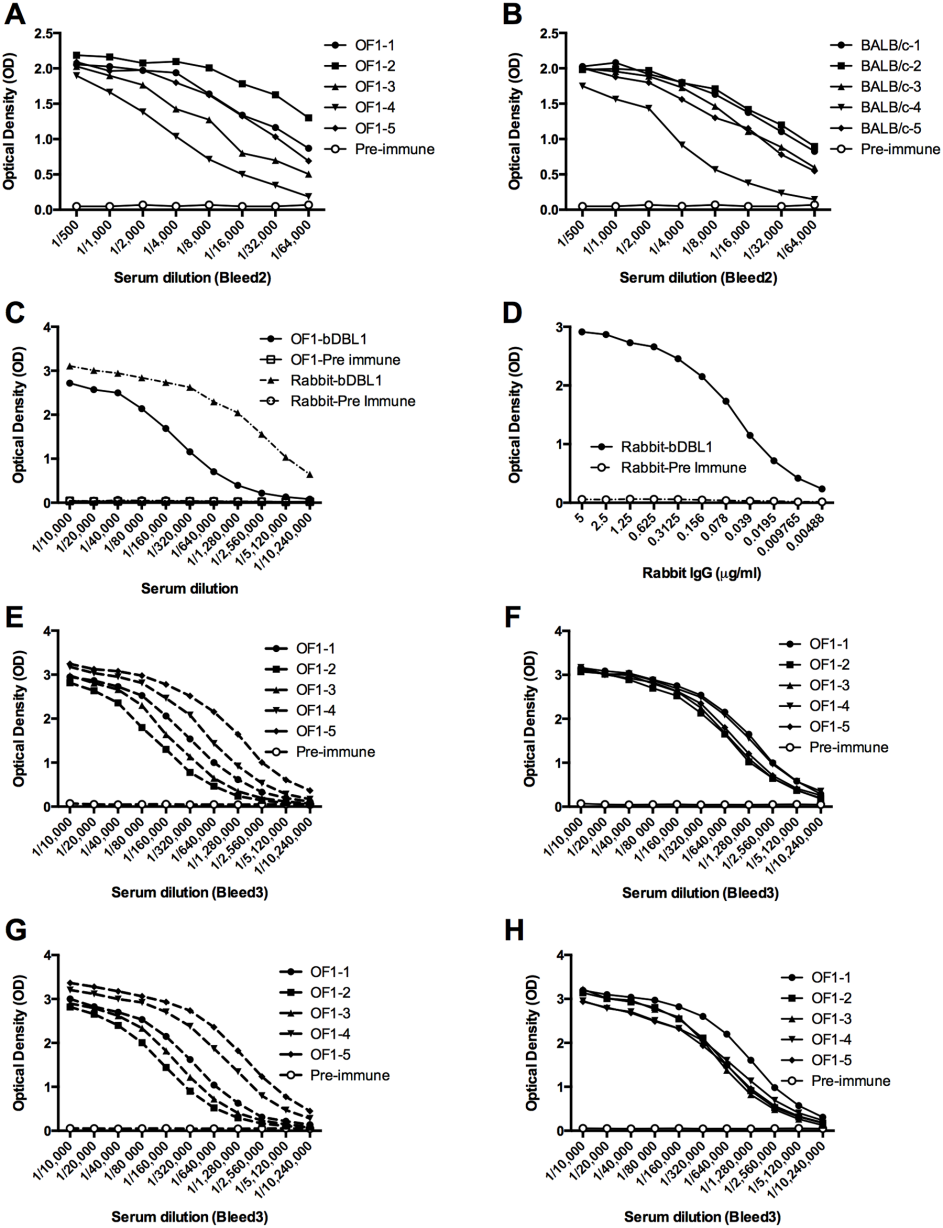
Influence of the expression system on the antibody response of animals immunised with PfEMP1-VarO DBL1 recombinant constructs. **(A and B)** Titration curves of individual bleed 2 sera collected from five outbred (OF1-1 to -5) (**A**) or five inbred (BALB/c-1 to -5) mice immunised with bDBL1 (**B**) on the immunising antigen (bDBL1). OF1 mice responded poorly. **(C)** Titration curves of serum from a rabbit immunised with bDBL1 compared to the pool of bleed 4 sera from OF1 mice immunised with bDBL1; antigen used in the ELISA: bDBL1. **(D)** Titration curve of rabbit IgG purified from the serum shown in (**C)**; antigen used in the ELISA: bDBL1. **(E to H)** Cross-checking on the homologous and heterologous expression product: titration curves of individual bleed 4 sera from OF1 mice immunised with bDBL1 on bDBL1 **(E)** and eDBL1 **(G)**; titration curves of individual bleed 4 sera from OF1 mice immunised with eDBL1 on eDBL1 **(F)** and bDBL1 **(H)**.

Two injections with bDBL1 were sufficient to elicit an antibody response in OF1 and BALB/c mice, although titres were dispersed over a wide range. After three injections, all inbred and outbred animals produced high antibody titres, although titres remained scattered particularly in OF1 mice (Fig 3A and 3B, respectively). The recombinant bDBL1 was highly immunogenic in the rabbit as well, with 50% titres of 1/2,560,000 and endpoint titres below 1/10^7^ for the serum after 4 doses (Fig 3C), and less than 0.5 ng.mL^-1^ for purified IgG (Fig 3D). These responses are higher responses than those observed in mice (Fig 3C).

**Fig 4:**
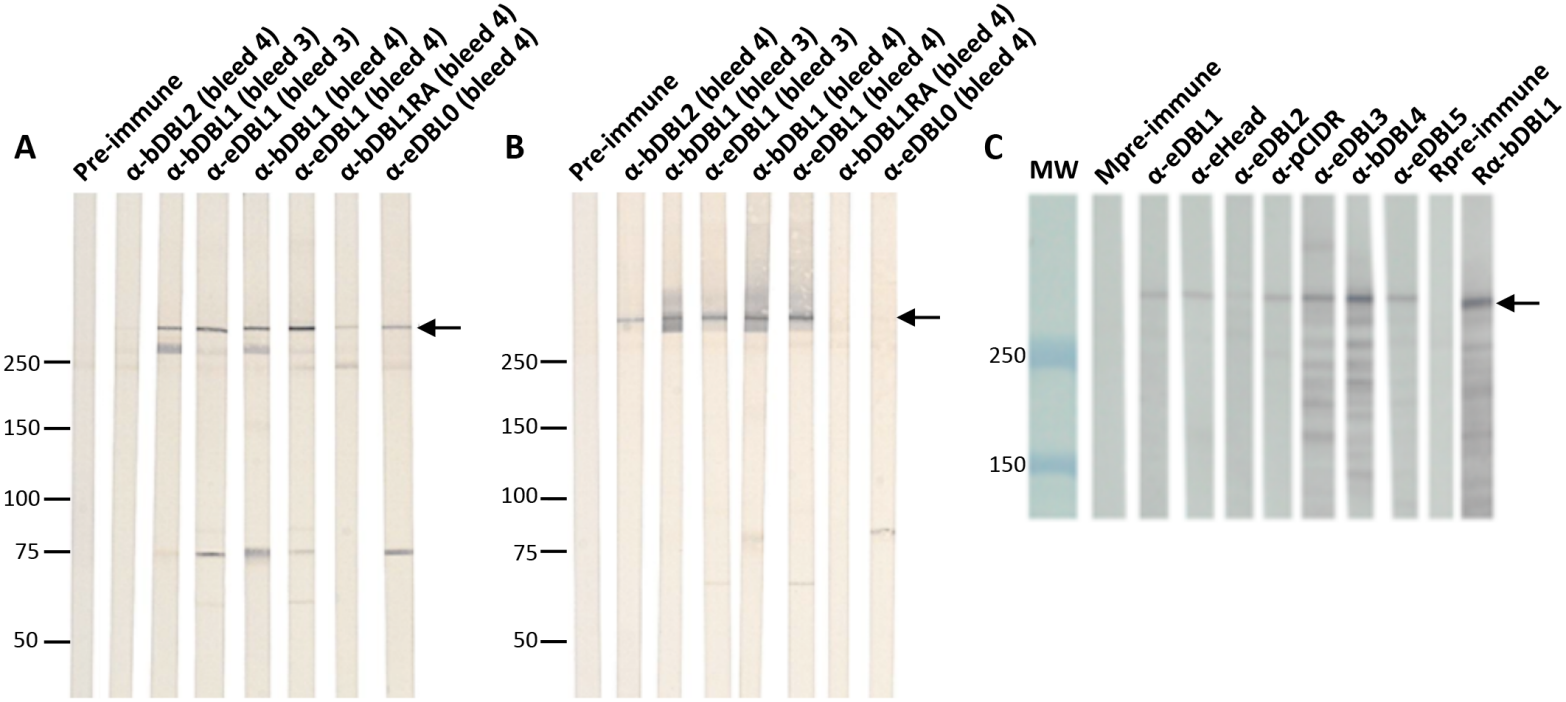
Immunoblot reactivity of antisera to PfEMP1-VarO-derived recombinant domains on Palo Alto VarO parasite extracts. Immunoblots were prepared from Palo Alto total extracts separated under reducing (**A, C**) or non-reducing conditions (**B**). Strips from immunoblots **(A)** and **(B)** were reacted simultaneously (2 strips in a given serum) with individual sera (dilution 1/200) (see Materials and Methods) as follows: lane 1: a representative mouse pre-immune serum (all were negative), lane 2: anti-bDBL2 (bleed 4), lane 3 anti-bDBL1 (bleed 3), lane 4 anti-eDBL1 (bleed 3); lane 5: anti-bDBL1 (bleed 4), lane 6 anti-eDBL1 (bleed 4), lane 7: anti-eDBL1RA (bleed 4); lane 8: anti-eDBL0 (bleed 4). (**C**) lane 1: a representative mouse pre-immune serum (idem), lane 2: anti-eDBL1, lane 3: anti-eHead, lane 4: anti-eDBL2, lane 5: anti-pCIDR, lane 6: anti-eDBL3, lane 7: anti-eDBL4, lane 8: anti-eDBL5, lane 9: pre-immune rabbit; lane 10: rabbit anti-bDBL1.

Inbred and outbred animals produced high titres of antibodies reacting with the parasite PfEMP1-VarO. Antisera to bDBL2 reacted with the non reduced PfEMP1-VarO only (an example is shown revised Fig 4A and 4B, lane 2). In contrast, individual polyclonal sera to bDBL1 reacted with endogenous PfEMP1-VarO in both reduced and non reduced parasite extracts. This was observed for sera collected after three and four injections (idem revised Fig 4A and 4B, lanes 3 and 5, respectively).

**Fig 5:**
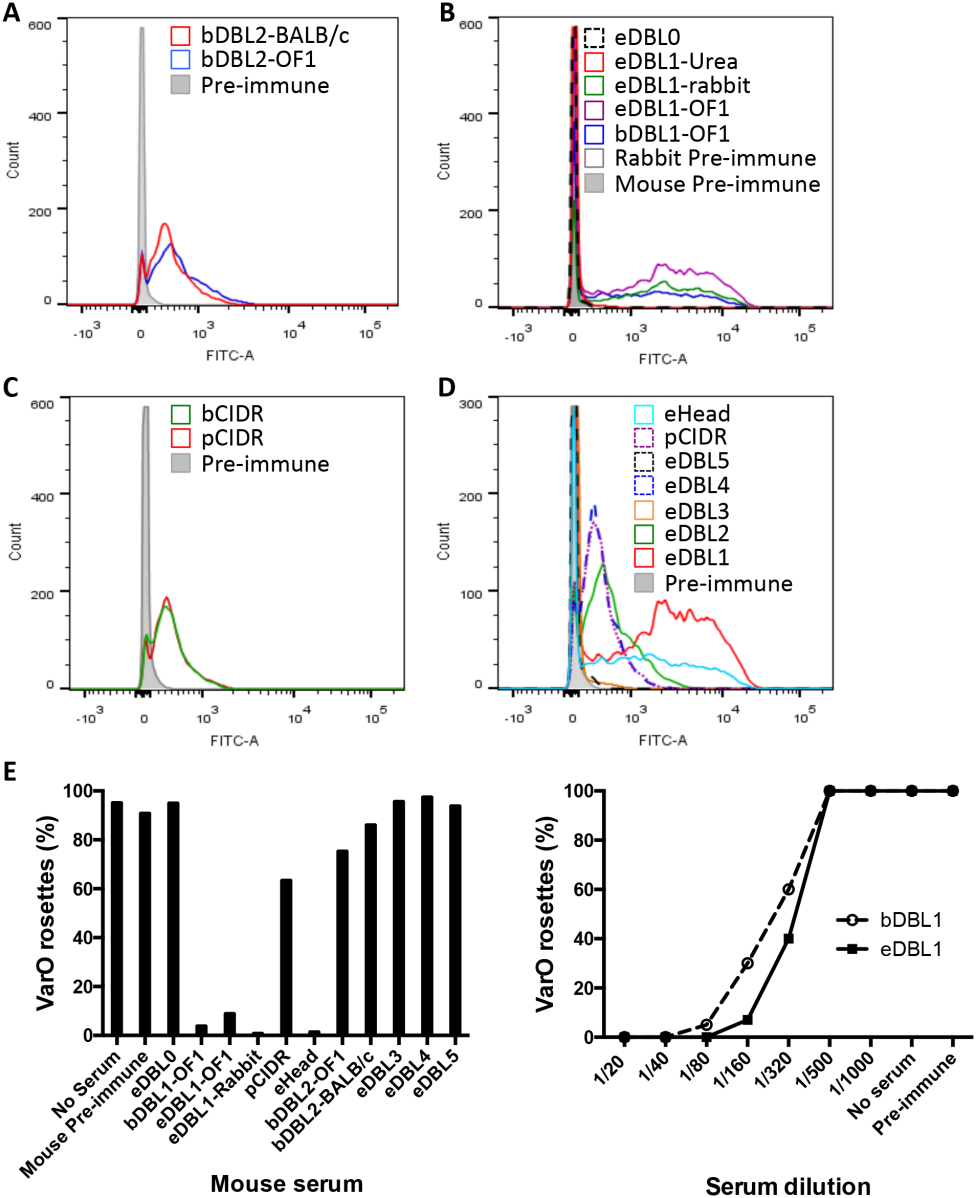
Surface reactivity and functionality of antibodies elicited by PfEMP1-Var0 derived recombinant constructs. Surface immunofluorescence of the monovariant Palo Alto 89F5 VarO parasites with sera raised to the recombinant domains and analysed by FACS. **(A)** Representative profiles of BALB/c and OF1 mice immunised with bDBL2. (**B)** Representative profiles of animals immunised with different DBL1 constructs as indicated. **(C)** Representative profiles of mice immunised with CIDR constructs as indicated. (**D)** Comparative profiles of pools of sera from mice immunised with PfEMP1-VarO derived domains as indicated. **(E)** Disruption capacity of Palo Alto VarO rosettes (see Materials and Methods for details). Left panel: sera from animals (at a 1/20 dilution) immunised with PfEMP1-VarO derived domains as indicated were tested for their capacity to disrupt rosettes; right panel: titration curves of the rosette-disrupting capacity of mouse sera raised to eDBL1 or bDBL1 as indicated.

All animals produced surface-reacting antibodies (examples are shown Fig 5A and 5B). Overall, 13/15 outbred animals immunised with bDBL1 had potent rosette-inhibiting and—disrupting antibodies (Fig 5E shows one example; see also S1 Fig). Rosette disruption was marginal with the anti bDBL2 sera (idem Fig 5E; Table 2).

**Table 2:** Reactivity of antibodies elicited after four immunisations with recombinant PfEMP1-VarO domains assessed using five readouts.

Antigen	Animals immunised	ELISA		Immunoblot VarO extract		VarO Surface reactivity	VarO rosette inhibition^§^	VarO rosette disruption^§§^
		End point titre^#^	50% titre^#^	Reduced	Non reduced	End point titre	Dilution 1/20	Dilution 1/20
eDBL0	5 BALB/c	625,000 *	25,000*	+	-	< 1/20	> 99	99.9 ± 5.7
	5 OF1	< 1/625,000*	< 1/625,000*	+	-	< 1/20	ND	ND
bDBL1	5 OF1	< 10,204,000	160,000	+	+	1/8,000	< 3	3.8 ± 0.7
	Rabbit serum	< 10,204,000	2,560,000	+	+	< 1/8,000	< 5	2.5 ± 2.4
	Rabbit IgG	< 4.8 ng.mL^-1^	78 ng.mL^-1^	+	+	5 μg.mL^-1^	<5 (10 μg.mL^-1^)	2.6 ± 1.5 (20 μg.mL^-1^)
eDBL1	5 OF1	< 10,204,000	640,000	+	+	< 1/8,000	< 5	12.1 ± 10.4
eDBL1RA^a^	5 BALB/c	51,200*	400*	+	-	< 1/20	> 95	10099.3 ± 5.7
bCIDR	6 BALB/c	128,000	25,000	-	+	1/1000	ND	ND
pCIDR	5 OF1	< 1,024,000	32,000	+	+	1/500	< 35	77.4 ± 19.8
bDBL2	6 BALB/c	< 204,800	25,600	-	+	1/1000	ND	92.4 ± 6.5
	6 OF1	< 204,800	51,200	-	+	1/4000	ND	84.5 ± 4.8
eDBL2RA^a^	5 OF1	80,000	20,000	+	-	< 1/20	> 95	10098.7 ± 4.3
eDBL3	7 OF1	< 512,000	32,000	+	faint	< 1/20	> 95	102.1 ± 2.9
eDBL4	7 OF1	< 512,000	32,000	+	+	1/2000	> 95	102.8 ± 4.1
eDBL5	6 OF1	< 10,240,000	640,000	+	-	< 1/20	> 95	101.6 ± 1.8
eHead	7 OF1	< 512,000	64,000	+	+	1/8,000	< 5	6.0 ± 5.0

^#^ Mean of the endpoint titre (mean pre-immune control ±3SD) or 50% titre (mean 50% OD value—OD pre-immune) on the antigen used for immunisation unless indicated

^**§**^ Percent rosettes relative to the pre-immune control.

^§§^ Percent rosettes relative to the pre-immune control (mean ± SD), at least 3 independent experiments were performed.

* Antigen used in ELISA: eDBL1;

^a^ RA: Reduced and Alkylated antigen;

ND: not determined.

We conclude that baculovirus constructs are immunogenic in inbred and outbred mice as well as rabbits and that three to four doses are needed to obtain a homogeneous antibody response, with high ELISA titres and high titres of antibodies reacting with endogenous PfEMP1-VarO on the iRBC surface. This immunisation regimen was used for all subsequent investigations.

### Influence of the expression system

We next compared immunogenicity of the soluble NTS-DBL1α construct expressed in the baculovirus/insect cells (bDBL1 used above) or in *E*.*coli* as Maltose-Binding fusion protein followed by factor Xa excision of the recombinant domain [19] (eDBL1). The two systems were shown to produce a functional recombinant protein that binds red blood cells [16]. Both proteins induced similar, high ELISA titres (50% titre: 1/640,000) after four doses in outbred mice (Fig 3E and 3F; Table 2). However, the response to the *E*. *coli* construct was more homogeneous than the response induced by the baculovirus product. Three independent immunisation experiments in outbred mice generated essentially identical results (data not shown). Cross-checking by ELISA on the heterologous expression construct indicated similar titres for anti-bDBL1 antisera on bDBL1 and eDBL1 constructs (Fig 3E and 3G, respectively); the same was observed for the anti-eDBL1 antisera (Fig 3F and 3H, respectively). This indicated that although immunogenicity of bDBL1 and eDBL1 differed, antigenicity was not markedly different.

Immunoblot reactivity was somewhat influenced by the expression system of the construct. Although antisera to bDBL1 and eDBL1 strongly reacted with PfEMP1VarO, the bDBL1 antisera (cross-)reacted on reduced extract with an approximately 270 kDa band, which was not recognised by eDBL1 antisera (Fig 4A lanes, 3 and 5 vs. 4 and 6). A similar difference was observed for reactivity with high molecular mass proteins on non reduced extracts (Fig 4B lanes, 3 and 5 vs. 4 and 6). No such a (cross)-reactivity was observed with rabbit sera raised to bDBL1 (Fig 4C lane 10). The significance of these different reactivity profiles is unclear and it was not explored further.

The antisera strongly reacted with the surface of VarO-infected red cells (Fig 5B). Surface IFA titres did not vary much from animal to animal. The anti-bDBL1 and eDBL1 antisera showed no difference in surface IFA titres (Table 2). A strong rosette-inhibition and rosette-disrupting response was observed in 5/5 mice immunised with eDBL1 and bDBL1 (Fig 5E, S1 Fig and Table 2). These data show that the eDBL1 and bDBL1 constructs potently induce functionally important antibodies. Both constructs are antigenically and immunogenically similar, though not identical. In particular, eDBL1 elicited a more homogeneous response in outbred mice.

### Immunogenicity of downstream DBL recombinant domains

We assessed immunogenicity of CIDRγ, which was produced as soluble protein in *Pichia pastoris* and baculovirus expression systems. Importantly, the *Pichia pastoris* pCIDR, unlike the pDBL1 construct, was modestly reactive with non immune human sera (mean OD ± SD, 0.182 ± 0.093) (Fig 1B). It induced high ELISA titres after three immunisations in 4/5 mice and in 5/5 outbred mice after the 4^rth^ immunisation (S2 Fig, and Table 2). The antibodies raised against pCIDR reacted on immunoblot with both reduced and non reduced extracts of Palo Alto VarO (an example is shown Fig 4C, lanes 5).

The longer baculovirus bCIDR construct was produced in low yields and immunisations were carried out by injecting 3 μg protein per dose rather than the standard 10 μg dose. Of six BALB/c mice immunised, only two responded with relatively low antibody titres (50% titre 1/6,400 and 1/50,000), probably reflecting use of a sub-optimal dosing. The antibody produced reacted with the VarO-iRBC surface (Fig 5C) and displayed moderate rosette-disrupting activity (Fig 5E) (Table 2).

The NTS-DBL1α-CIDRγ domain produced in *E*. *coli* (eHead) bound quite efficiently to uninfected RBC [16]. The antibody response of outbred mice (N = 7) was qualitatively similar to the response induced by eDBL1 but tended to be quantitatively different, with lower ELISA titres (S3A Fig), lower FACS surface reactivity (Fig 5D) but higher rosette disruption than usually obtained with sera to eDBL1 (Fig 5E and Table 2).

The downstream domains DBL3γ, DBL4ε and DBL5ε produced in *E*. *coli* (eDBL3, eDBL4 and eDBL5, respectively) induced ELISA antibody titres in the same range as those induced by pCIDR or eHead and responses were homogeneous (S3B–S3G Fig).

All sera reacted by immunoblot (a representative example for each domain is shown Fig 4C, lanes 3–8). Antisera to eDBL4 reacted with the VarO-iRBC surface but no surface reactivity could be recorded for antisera raised to eDBL3 or eDBL5 (Fig 5D and Table 2). None of the antisera had detectable rosette-inhibition nor rosette-disruption capacity at the dilution tested (Fig 5E, left panel and Table 2).

### Consequences of denaturation and reduction/alkylation

**Fig 6:**
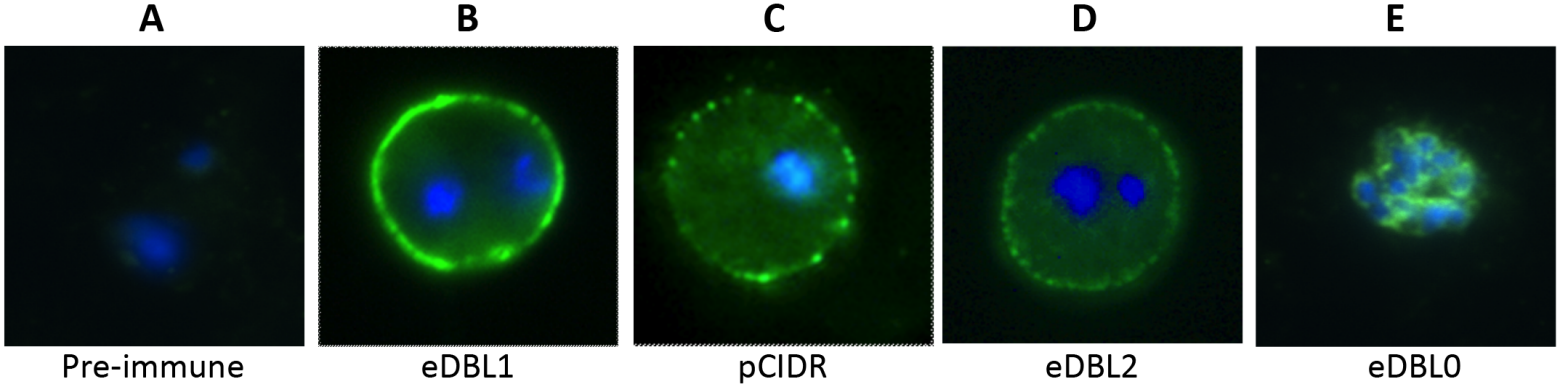
Immunofluorescence assay with live infected or formalin-fixed PaloAlto VarO erythrocytes. Live Palo Alto VarO mature infected erythrocytes (pigmented trophozoites and schizonts) **(A-D)** or formalin-fixed cultures **(E)** were incubated with mouse antisera against PfEMP1-VarO-derived recombinant domains at 1/200 dilution, and bound antibodies visualised using Alexa Fluor 488-labelled goat anti-mouse IgG (Invitrogen) at 1/1,000 dilution and nuclei were labelled with Hoechst 33342 dye at 1/1,000. Typical punctate surface staining of the membrane of the infected erythrocyte (green) was observed with antisera raised to bDBL1, eDBL1, pCIDR, eDBL2, bDBL2, eDBL4 and eHead (representative examples are shown here in **(B-D)** as indicated. Antisera raised to eDBL0, which failed to react with the erythrocyte surface, reacted with a perinuclear image on formalin-fixed Palo Alto VarO parasites **(E)**. Pre-immune sera to formalin-fixed parasites were negative (not shown). The parasite nuclei are stained with DAPI (blue). Slides were viewed with a 100x objective using a Leica CTR5000 fluorescent microscope (35).

Construct eDBL0, devoid of the NTS region, was produced as inclusion bodies, solubilised in 3 M urea, 10 mM DTT and injected in a denatured form. It was quite immunogenic, as indicated by high ELISA reactivity with the native eDBL1 and bDBL1 constructs (S4A and S4B Fig, Table 2). The individual response of BALB/c mice was somewhat scattered, with 50% ELISA titres ranging from 1/25,000 to 1/125,000 on eDBL1, and even more scattered when assessed against bDBL1, with 50% ELISA titres ranging from 1/5,000 to 1/75,000. The protein was more immunogenic in OF1 outbred mice (50% ELISA titres consistently above 1/625,000 for 4/5 animals, data not shown), i.e. in the same range as outbred mice immunised with the native eDBL1 or bDBL1 (Table 2). The sera reacted with PfEMP1-VarO on immunoblots of reduced Palo Alto iRBCs, but failed to react with the non—reduced extract (Fig 4A and 4B, lane 8). The sera also failed to react by surface IFA (Fig 5B) and to prevent or interfere with VarO rosetting (Fig 5E and Table 2). Interestingly, the sera reacted by IFA on fixed parasite smears, producing a perinuclear image, consistent with a reaction with the ER-enclosed, unfolded protein (Fig 6). We conclude that this denatured N-terminal domain, truncated of its NTS region induces antibodies reacting with PfEMP1 epitopes that are not displayed on the iRBC surface.

We next investigated the consequences of reduction and alkylation on immunogenicity of eDBL1 and eDBL2 (eDBL1RA and eDBL2RA). Both proteins elicited low antibody titres (S4C and S4D Fig). Sera reacted with Palo Alto 89F5 VarO extracts under reduced conditions but failed to react with non-reduced protein extracts (representative results are shown in Fig 4A and 4B, lane 7 for eDBL1RA), failed to react with the VarO-irRBC surface and did not inhibit nor disrupt rosettes (Fig 5B and 5E, Table 2), indicating that production of antibodies to surface-displayed epitopes depends on the presence of native disulfide bonds in the immunising domain.

### Inter-domain cross-reactivity

**Fig 7:**
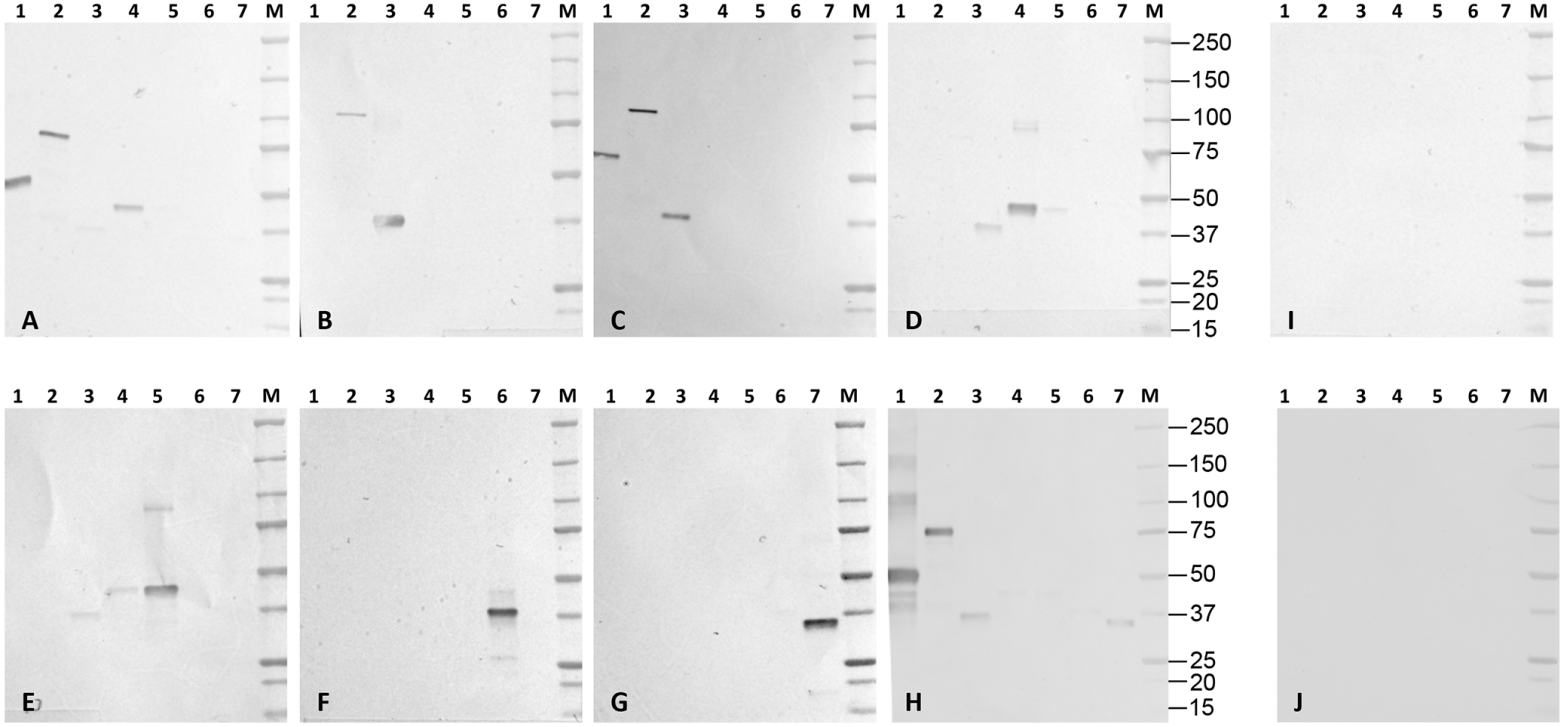
Specificity of the antisera raised to PfEMP1 VarO-derived recombinant domains assessed by immunoblot on homologous and heterologous recombinant domains. Recombinant domains (50 ng each) were separated on 4–12% SDS gels and immunoblotted. Ten immunoblots were prepared in parallel and loaded as follows: eDBL1 (lane 1), eHead (lane 2), pCIDR (lane 3), bDBL2 (lane 4), eDBL3 (lane 5), eDBL4 (lane 6) and eDBL5 (lane 7). Blots were incubated with anti-sera against individual domains (diluted 1/ 500). **(A)**: OF1 mouse against bDBL1; **(B)**: OF1 mouse against pCIDR; **(C)**: OF1 mouse against e-Head; **(D)**: OF1 mouse against bDBL2; **(E)**: OF1 mouse against eDBL3; **(F)**: OF1 mouse against eDBL4; **(G)**: OF1 mouse against eDBL5; **(H)**: rabbit against bDBL1; **(I)**: a pool of mouse pre-immune sera and **(J)**: rabbit pre-immune serum.

**Fig 8:**
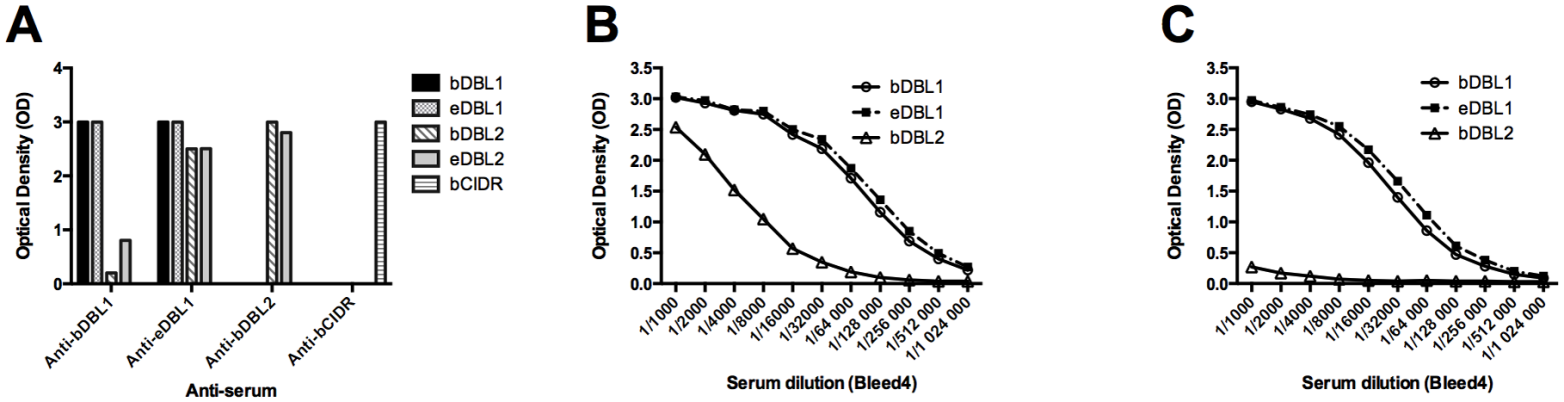
Cross-reactivity of mouse sera raised to DBL1 constructs with bDBL2. ELISA assays of OF1 mouse antisera against recombinant antigens. (**A**) Reactivity of bleed 4 antisera against bDBL1, eDBL1, bDBL2 and bCIDR (dilution 1/10,000) with the cognate and heterologous antigens. Symbols for antigens are indicated on the right. (**B and C**) Titration curves of bleed 4 sera against bDBL1 **(B)** or eDBL1 **(C)** on eDBL1, bDBL1 and bDBL2 antigens. Symbols for antigens are indicated on the right.

When tested by immunoblot on the cognate and heterologous recombinant VarO domains, most sera essentially displayed domain-specific reactivity (Fig 7). However, there was some faint reaction of rabbit anti-bDBL1 with pCIDR and eDBL5 (panel H), of mouse anti-eDBL3 with pCIDR and eDBL2 (panel E) and mouse anti-bDBL2 with eDBL3 (this latter case probably reflecting the 22 amino acid sequence overlap of the DBL2 and DBL3 constructs—alignment of the various sequences is shown in S5 Fig). A stronger cross-reaction of outbred mouse anti-eDBL1 antisera with eDBL2 was observed (panel A). ELISA confirmed that antibodies raised in outbred mice to eDBL1 cross-reacted with bDBL2 and eDBL2. Antisera to bDBL1 cross-reacted less strongly with bDBL2 and eDBL2 (Fig 8). Such cross-reactivity was not observed with antibodies raised against these proteins in BALB/c mice (data not shown). Moreover it was not reciprocal, as antisera to either bDBL2 or eDBL2 failed to react with the DBL1 constructs (Fig 7 panel D and Fig 8). Thus, immunoblot and ELISA data suggest that eDBL1 and e/bDBL2, which have 27–28% protein sequence identity, share some epitopes that elicit in some animals cross-reacting antibodies when presented by the DBL1 constructs.

## Discussion

Vaccination with recombinant antigens necessitates optimising a large number of parameters, including expression system, amount of protein injected, immunisation schedule, number of doses, adjuvant and delivery route. Only some of these parameters were investigated here, as we wanted to explore inter-individual variation in outbred and inbred mice using five readout assays. The scatter of antibody titres observed after the second dose, was substantially reduced after the third dose, resulting in homogeneous profiles both on the immunising antigen and on the various parasite-dependent assays. Importantly, outbred OF1 mice responded by a consistent production of high ELISA titres and good titres of antibodies reacting with the parasite PfEMP1-VarO protein in the parasite iRBC context. This shows good prospects for an effective vaccination strategy against rosetting, as an important prerequisite is to induce a potent, functionally active immune response targeting the iRBC surface in a large proportion if not all, of vaccinees.

Immunogenicity of the native individual PfEMP1-VarO domains was high whatever the expression system used. This is in line with the recent multilaboratory study on Var2CSA domains [31], although contrasting with other published studies [22, 24]. However, both antigenicity and immunogenicity of PfEMP1-VarO domains were finely influenced by the expression system. The bDBL1 and eDBL1 constructs appeared no different with regard to antigenicity, being interchangeable as coating antigens in the ELISA and similarly recognised by immune human sera. Antigenicity of pDBL1 was different, with high background reactivity with non-immune human sera; pDBL1 was therefore discarded from our immunogenicity study. Likewise, non-immune human sera reacted differently with bCIDR1 and pCIDR1. With regard to immunogenicity, a wider scatter of antibody titres was elicited by bDBL1 than by eDBL1. Both systems did however induce potent surface—reacting and rosette-blocking antibodies. Our data show that the *E coli* expression system used here (production as Maltose Binding fusion proteins followed by factorXa excision of the recombinant domain) provides adequate immunogens in large amounts and, as such, is a convenient expression system for single domains and the Head double domain.

ELISA titres elicited by most VarO domains were usually higher than reported so far for PfEMP1-derived antigens administered at a similar dose in Freund's adjuvant. For instance for DBL1, 50% titres in rabbits were 5-fold higher than in rabbits immunised with a similar dose of PfEMP1VarR29-DBL1 [14], at least 10-fold higher than the 50% titres observed in rabbits immunised with DBL1 domains from other rosette-forming PfEMP1 [10] and 100-fold higher than in goats and rats immunised against PfEMP1 DBL1 domains [30, 45]. Endpoint titres for all domains were higher than those usually reported for PfEMP1-Var2CSA individual domains injected in Freund's adjuvant [23, 24, 34], including studies that reported improved immunogenicity and higher ELISA titres [31, 32, 34]. Of note, immunisation with different batches of eDBL1, bDBL1 or bDBL2 induced quite similar ELISA titres and surface IFA reactivity, indicating reproducible immunogenicity in outbred animals. High ELISA titres were a hallmark of properly folded immunogens. Indeed, titres observed in mice immunised with the reduced-alkylated antigens were several log units lower than those elicited by the non-reduced antigens. ELISA titres were poorly related with biological activity such as surface reactivity, differing from some studies on Var2CSA-derived immunogens [34]. Thus, although ELISA titres were poorly informative with regard to antibody functionality, they provided a robust profile characteristic of the immunogen.

All recombinant PfEMP1-VarO domains elicited strong immunoblot reactivity with the parasite-derived PfEMP1-VarO protein and, apart from eDBL3 and eDBL5, all constructs elicited surface-reactive antibodies. Strong MFI values were observed by FACS and here as well, there was no major influence of the expression system, contrasting with reported data for PfEMP1-Var2CSA [24]. The titres of iRBC surface reactivity of anti-DBL1 antisera (above 1/8,000) compare well with titres reported for various PfEMP1 domains of rosette-forming parasites [10, 14]. Titres were lower for the downstream domains, yet higher than the surface reactivity reported for various PfEMP1-derived domains [22, 30, 32, 34, 46–48]. The reason for the failure of eDBL3 and eDBL5 domains to elicit surface-reactive antibodies is unclear. It is possible that these domains are buried within the iRBC surface-displayed molecule. An alternative is they were not expressed with the native fold. The eDBL3 and eDBL5 CD spectra did not depart from the CD spectra of the other domains [16] indicating absence of major biophysical differences. Moreover, both were readily recognised by sera from humans living in endemic areas (Fig 1 and manuscript in preparation). This indicates that they display epitopes exposed to the immune system during natural parasite infection, but does not preclude that they do not display some surface-exposed epitopes. As we could not assign any specific binding characteristics to eDBL3 and eDBL5, we cannot ascertain that the recombinant antigens are functional. More work is needed to understand surface display of each individual PfEMP1-VarO domain. It is worth noting however, that this is not unprecedented as several studies reported that some domains of PfEMP1-Var2CSA failed to induce surface reactive antibodies [22, 24, 25, 49].

Several lines of evidence indicate that iRBC-displayed surface epitopes are disulfide bond-dependent, conformational epitopes. VarO-iRBC surface reactivity was correlated with reactivity on immunoblot of non-reduced parasite extracts and some surface-reacting polyclonal sera (against bCIDR or anti-bDBL2) failed to react on immunoblots of reduced parasite antigens. In contrast, the anti-eDBL0 antisera failed to react on immunoblots of unreduced parasite antigens and failed to react with the VarO-iRBC surface. This indicates that the urea-denatured protein, which moreover lacks the functionally essential NTS domain [19] does not present surface-displayed epitopes. Likewise, antisera raised to the reduced-alkylated eDBL1RA or eDBL2RA failed to react with the iRBC surface and failed to react on immunoblot of unreduced parasite extracts. This is in line with results showing that antisera against denatured iodoacetamide treated-Var2CSA DBL5 failed to react with the surface of Var2CSA expressing parasites, while antisera to the native domain did so [47]. The crystal structure of VarO-DBL1 [19] and VarO-Head [16] shows numerous disulfide bonds that shape protein surface areas with nonlinear sequence fragments. It is thus no surprise that surface epitopes depend on the proper formation of disulfide bonds. It is unlikely that such epitopes will be mimicked by synthetic peptides, although some success has been reported in rats immunised with a specific peptide sequence of subdomain 2 of PfEMP1-DBL1α [46].

Efficient cytoadherence inhibition/disruption was observed only with antibodies to DBL1 and to the Head domain, and inhibition/disruption by anti-CIDR and anti-DBL2 antibodies antibodies was modest. Thus, the capacity to elicit antibodies interfering with rosetting seems restricted to few PfEMP1-VarO domains and is a property of the RBC adhesion domain. This differs from the situation reported for the rosette-forming variant IT4-R19 (Var9) in which antibodies to NTS-DBL1 as well as antibodies to DBL2C2 potently reversed rosette formation and moreover antibodies to five individual domains inhibited rosette formation [14]. This discrepancy may reflect different sensitivity of the rosette inhibition and rosette disruption assay or intrinsic features of the adhesins, in particular different domain organisation as well as different cytoadherence properties. IT4-R19 (Var9) parasites form rosettes by binding to CR1 [37] but also bind to brain endothelial cells [38] and were reported to bind to CSA as well [50]. In contrast, PaloAlto VarO parasites appear to present a single cytoadherence phenotype rosette formation, which involves binding to the abundant ABO blood group antigen on uninfected RBCs and VarO-iRBC forming giant rosettes [9, 39] but do not bind to an array of endothelial receptors [51]. Whether these differences involve a different spatial organisation of the various PfEMP1 domains on the parasite surface requires further studies.

Cross-reactivity of the anti-eDBL1 antibodies (and to a much lesser extent of the anti-bDBL1 antibodies with bDBL2 and eDBL2 was unexpected as these domains have limited homology (27–28% identity) [39] and no known shared functionality [16]. Cross-reactivity was unidirectional as the anti-bDBL2 antibodies failed to react with both eDBL1 and bDBL1, suggesting that it is a property of DBL1-derived immunogens. Immunoblots indicated that anti-eDBL1 antibodies cross-reacted with the eDBL2 recombinant domain itself and not with putative contaminating molecule(s) (Fig 7). The rabbit antibodies raised against bDBL1 presented a different, faint immunoblot cross-reactive profile to the pCIDR and DBL5 domains. This suggests possible recognition of similar epitopes displayed by different domains. Cross-reactivity between domains of different types has been observed previously in the case of PfEMP1-IT4Var9 R29, as DBL4δ antibodies bound with high efficiency the NTS-DBL1α domain [14].

We confirm here that antibodies reacting with iRBC surface are readily and consistently produced in animals immunised with the DBL1 or Head domains from rosette-forming variants [10, 14, 45]. This differs from reports concerning PfEMP1-Var2CSA indicating substantial inter-animal variability [22, 25, 35] and poor relationship between receptor-binding capacity, induction of surface-reacting antibodies and induction of functionally active, adhesion inhibitory antibodies [32–35, 49, 52, 53]. As only DBL1-containing constructs elicited high amounts of rosette-disrupting antibodies, we tend to consider DBL1-containing constructs as prime vaccine candidates against rosetting, rather than "multiple extracellular domains of rosette-mediating PfEMP1 variants" as proposed by Ghumra et al [10].

The elevated titres of rosette-disrupting antibodies evidenced here markedly differ from our observations in endemic areas. Indeed, all children plasma tested in Senegal [39, 40] or Benin [54] lacked rosette-disrupting antibodies, although VarO-iRBC surface reacting antibodies were readily detected and with a high seroprevalence. This suggests that vaccination using functional DBL1 or Head domains may altogether harness natural immunity (surface reactivity) and implement an additional effector mechanism (rosette disruption). We did not test opsonisation of iRBC in immunised animals, although production of opsonising antibodies was shown to parallel the production of surface reacting antibodies against rosette-forming PfEMP1 domains [14]. In summary, the results reported here are promising for vaccine development and constitute a solid basis for future studies that will include dose-response and adjuvant studies, investigation of the breadth of the antibody response against a large panel of field isolates as well as of the duration of vaccine induced immunity, a critical issue for a vaccine in the era of malaria elimination.

## Supporting Information

S1 Fig Palo Alto VarO rosette disruption capacity of individual anti-bDBL1 and anti-eDBL1 mouse sera. Individual sera (bleed 4) from two groups of five OF1 mice each immunised with either bDBL1 or eDBL1 were tested for their capacity to disrupt Palo Alto VarO rosettes by adding the mouse serum (final dilution 1/20) to parasite cultures containing 10% human AB+ serum. A representative pre-immune mouse serum is shown for each series. Non-immune mouse sera were all negative.
(TIFF)

S2 Fig Four immunisations are needed to elicit a homogeneous response to pCIDR in outbred mice. Titration curves of individual bleed 3 (**A**) and bleed 4 (**B**) sera collected from five outbred (OF1-1 to 5) immunised with pCIDR and tested on the immunising antigen.
(TIFF)

S3 Fig Antibody responses against eHead, eDBL3, eDBL4 and eDBL5 assayed by ELISA on the immunising antigen. (**A**) Immunisation against eHead: titration curves of serum pools from bleed 2–5, showing that a high, sustained response was achieved after two inoculations of antigen. Immunisation against eDBL3 (**B, C**), eDBL4 (**D, E**) and eDBL5 (**F**, **G)**; titration curves of individual bleed 5 (**B, D, F**) and serum pools from successive bleeds as indicated (**C, E, G**)
(TIFF)

S4 Fig Antibody responses against denatured DBL1 and DBL2 domains. Titration curves of individual bleed 3 (**A**) and bleed 4 (**B**) sera collected from five BALB/c mice (BALB/c-1 to 5, same animal numbering in both graphs) immunised with eDBL0 in the presence of 3M Urea. The antigen used to coat the ELISA plates was bDBL1 (**A**) and eDBL1 (**B**). Titration curves of individual bleed 4 sera from outbred mice immunised with reduced-alkylated eDBL2RA assayed on eDBL2RA (**C**) or eDBL2 (**D**). Titration curves of individual bleed 4 sera from outbred mice immunised with reduced-alkylated eDBL1RA assayed on eDBL1RA (**E**) and eDBL1 (**F**).
(TIFF)

S5 Fig Alignment of the protein sequences of the cross-reacting domains. (**A**) DBL1/pCIDR/DBL5 alignment: DBL1 residues 1-437/ pCIDR residues 508-787/ DBL5 residues 2025–2321. (**B**) DBL3/pCIDR/DBL2 alignment: DBL3 residues 1220-1578/ pCIDR residues 508-787/ DBL2 residues 821–1242. (**C**) DBL2/DBL3 alignment: DBL2 residues 821-1242/ DBL3 residues 1220–1578. (**D**) DBL1/DBL2 alignment: DBL1 residues 1–437 / DBL2 residues 821–1242. (E) Alignment of all individual VarO domains expressed as recombinant proteins.
(DOCX)
