# Supplementary figures and images for: Immunogenicity of the Plasmodium falciparum PfEMP1-VarO Adhesin: Induction of Surface-Reactive and Rosette-Disrupting Antibodies to VarO Infected Erythrocytes
Source: PLoS One. 2015 Jul 29;10(7):e0134292. doi: 10.1371/journal.pone.0134292 (PMC4519321; doi:10.1371/journal.pone.0134292)

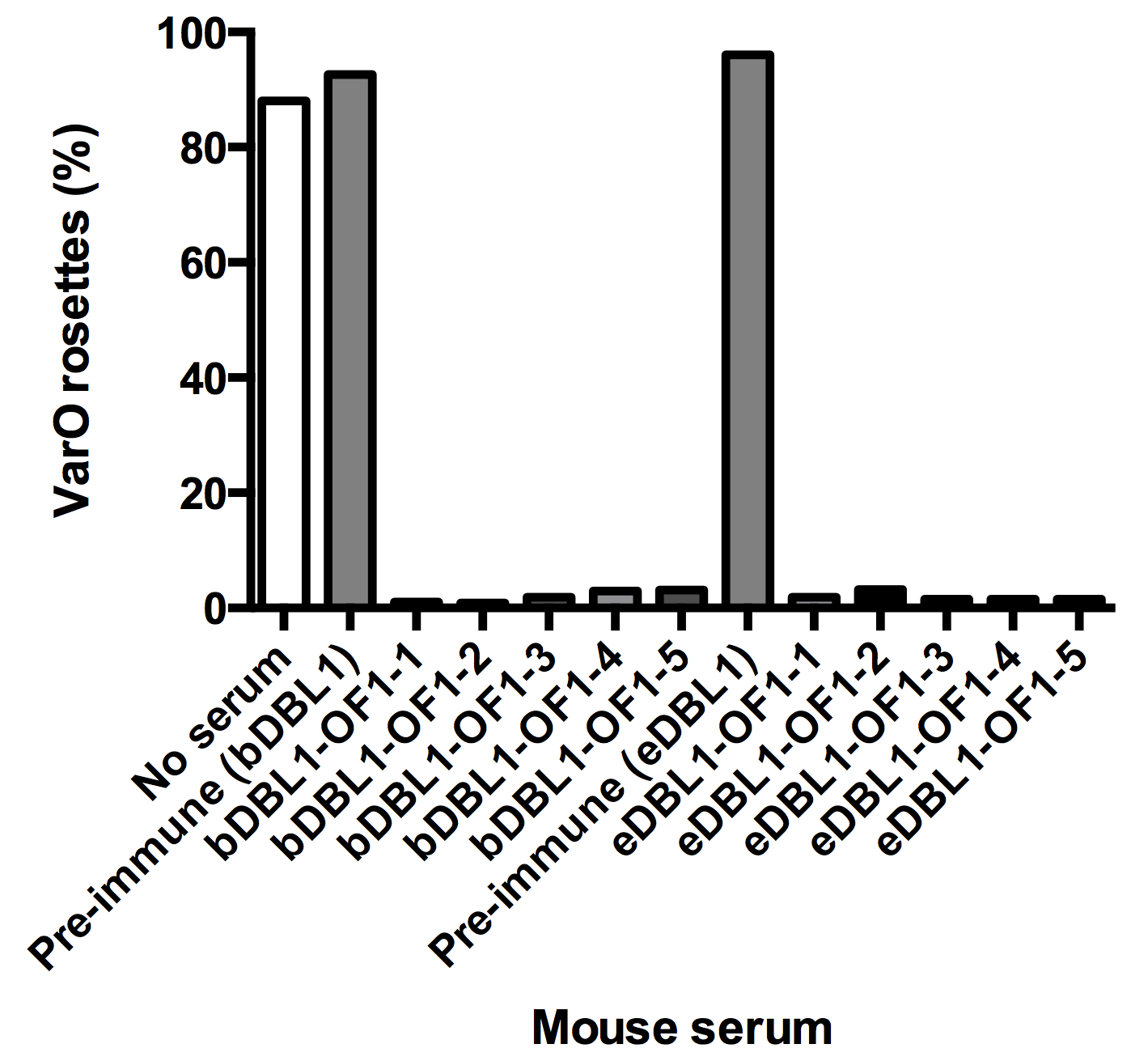

Supplement: S1 Fig — Individual sera (bleed 4) from two groups of five OF1 mice each immunised with either bDBL1 or eDBL1 were tested for their capacity to disrupt Palo Alto VarO rosettes by adding the mouse serum (final dilution 1/20) to parasite cultures containing 10% human AB+ serum. A representative pre-immune mouse serum is shown for each series. Non-immune mouse sera were all negative. (TIFF) [file pone.0134292.s001.tiff]

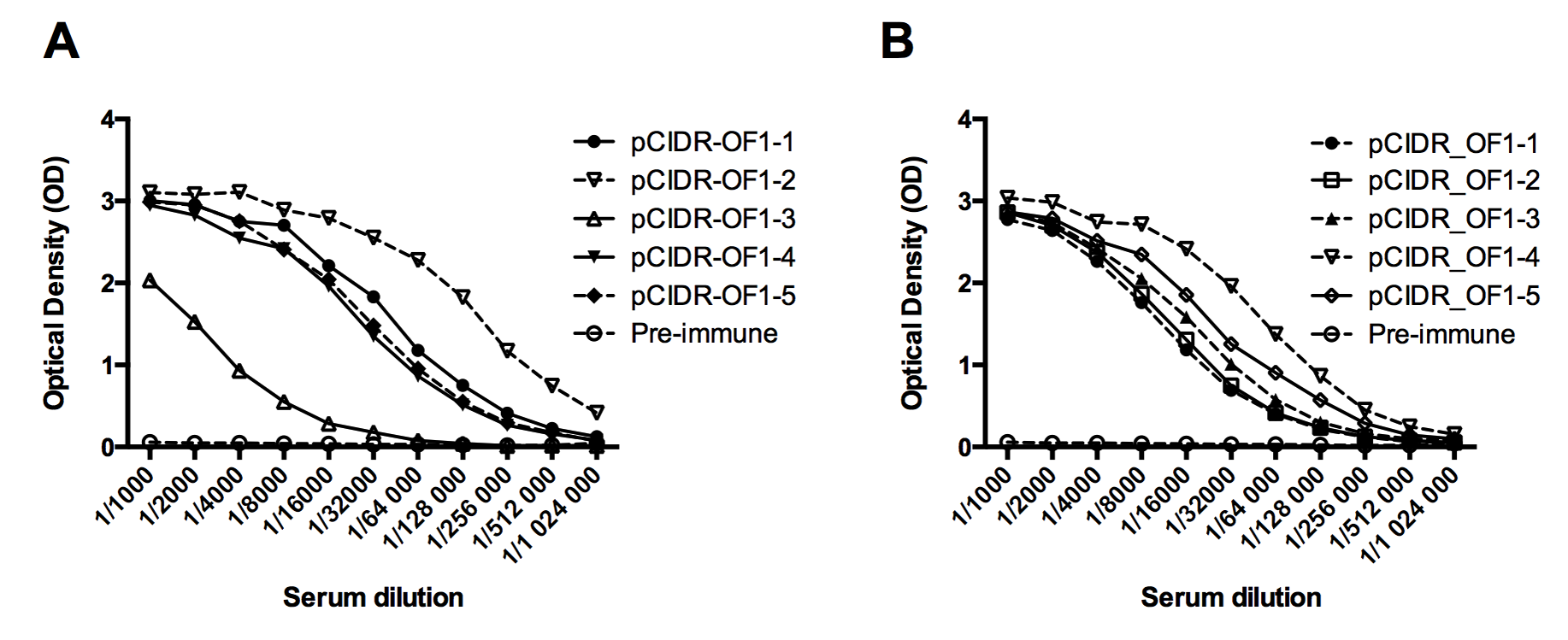

Supplement: S2 Fig — Titration curves of individual bleed 3 (A) and bleed 4 (B) sera collected from five outbred (OF1-1 to 5) immunised with pCIDR and tested on the immunising antigen. (TIFF) [file pone.0134292.s002.tiff]

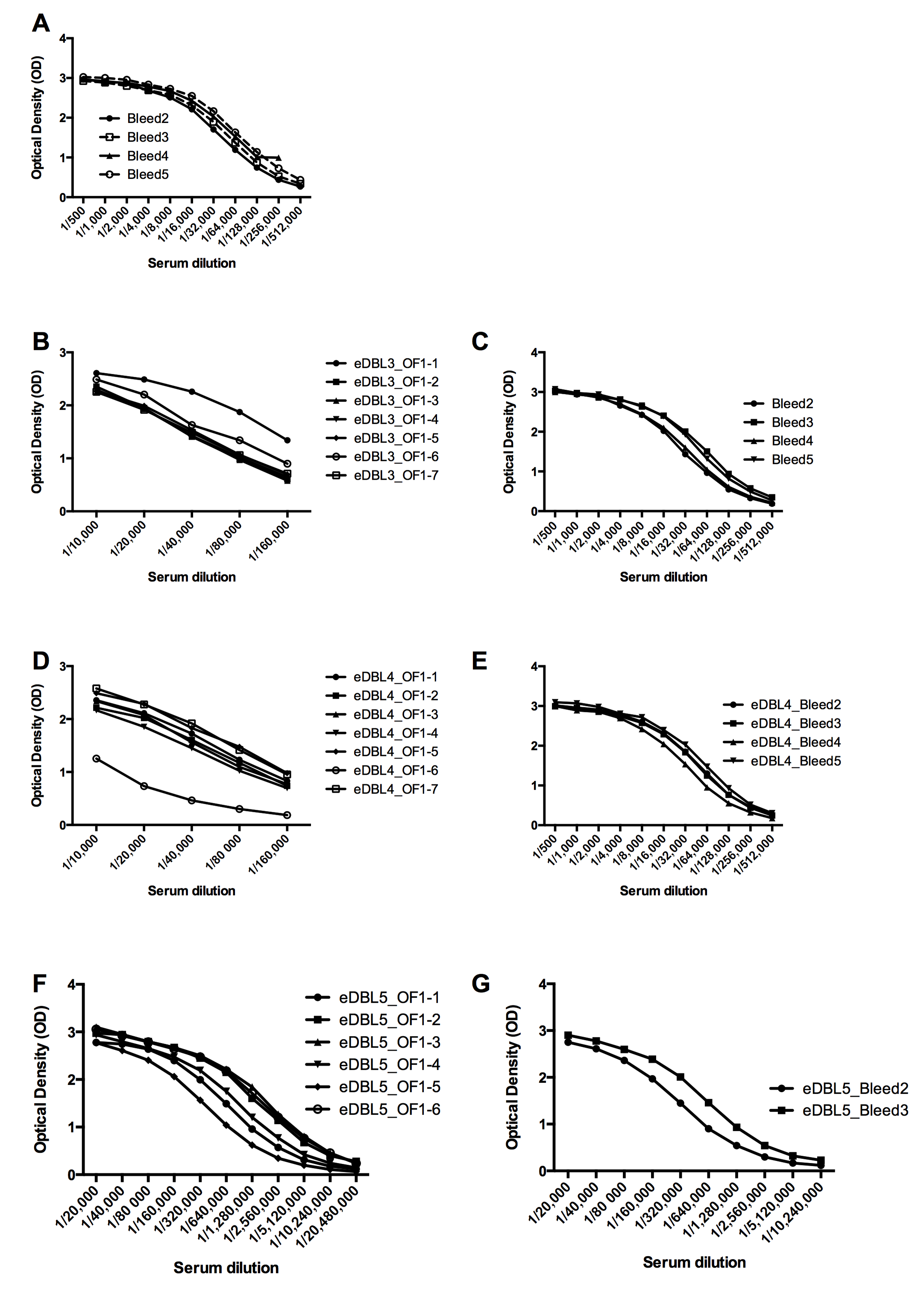

Supplement: S3 Fig — (A) Immunisation against eHead: titration curves of serum pools from bleed 2–5, showing that a high, sustained response was achieved after two inoculations of antigen. Immunisation against eDBL3 (B, C), eDBL4 (D, E) and eDBL5 (F, G); titration curves of individual bleed 5 (B, D, F) and serum pools from successive bleeds as indicated (C, E, G) (TIFF) [file pone.0134292.s003.tiff]

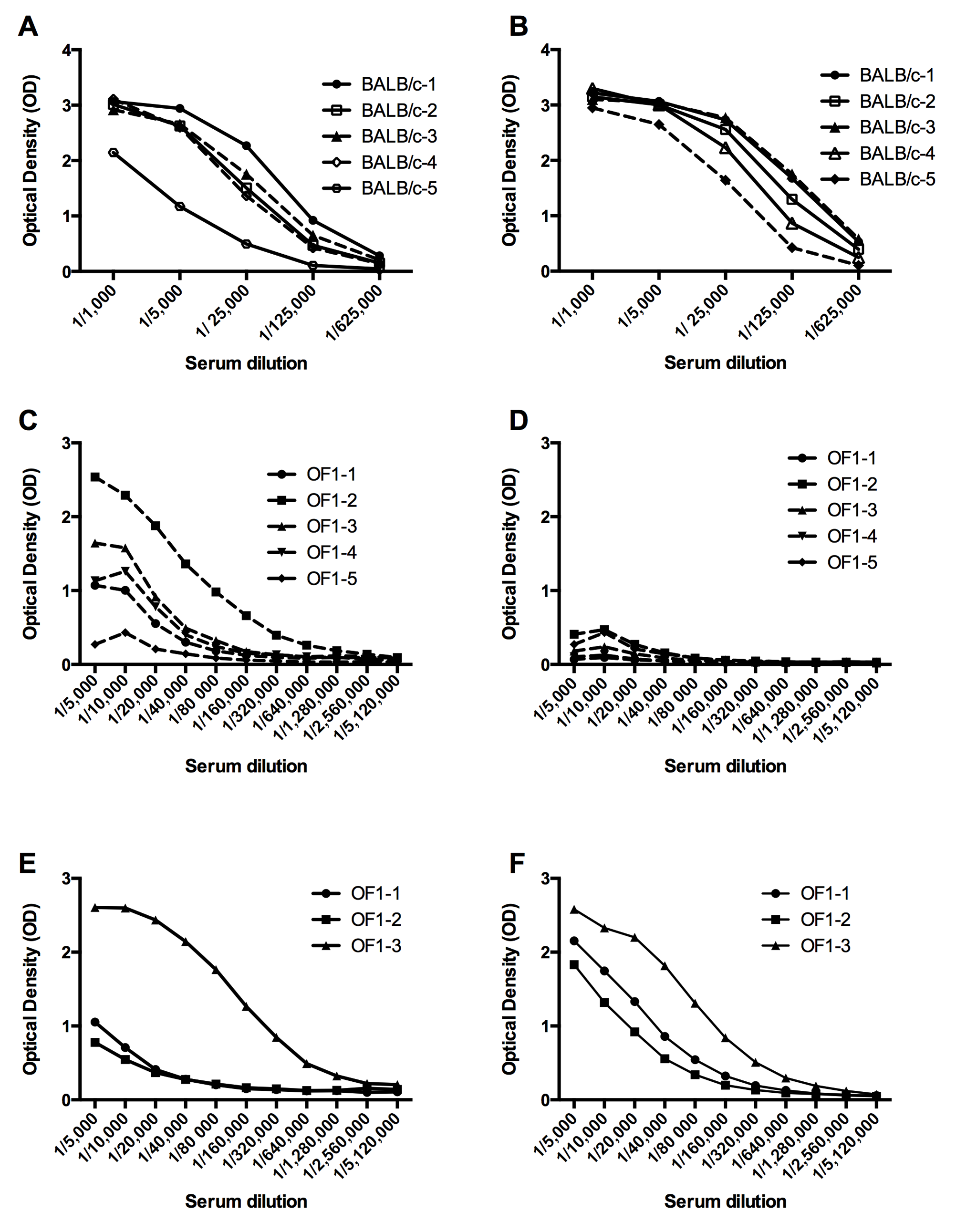

Supplement: S4 Fig — Titration curves of individual bleed 3 (A) and bleed 4 (B) sera collected from five BALB/c mice (BALB/c-1 to 5, same animal numbering in both graphs) immunised with eDBL0 in the presence of 3M Urea. The antigen used to coat the ELISA plates was bDBL1 (A) and eDBL1 (B). Titration curves of individual bleed 4 sera from outbred mice immunised with reduced-alkylated eDBL2RA assayed on eDBL2RA (C) or eDBL2 (D). Titration curves of individual bleed 4 sera from outbred mice immunised with reduced-alkylated eDBL1RA assayed on eDBL1RA (E) and eDBL1 (F). (TIFF) [file pone.0134292.s004.tiff]
