# Supplementary material for: Immunogenicity of the Plasmodium falciparum PfEMP1-VarO Adhesin: Induction of Surface-Reactive and Rosette-Disrupting Antibodies to VarO Infected Erythrocytes
Source: PLoS One. 2015 Jul 29;10(7):e0134292. doi: 10.1371/journal.pone.0134292 (PMC4519321; doi:10.1371/journal.pone.0134292)
Supplement: S5 Fig — (A) DBL1/pCIDR/DBL5 alignment: DBL1 residues 1-437/ pCIDR residues 508-787/ DBL5 residues 2025–2321. (B) DBL3/pCIDR/DBL2 alignment: DBL3 residues 1220-1578/ pCIDR residues 508-787/ DBL2 residues 821–1242. (C) DBL2/DBL3 alignment: DBL2 residues 821-1242/ DBL3 residues 1220–1578. (D) DBL1/DBL2 alignment: DBL1 residues 1–437 / DBL2 residues 821–1242. (E) Alignment of all individual VarO domains expressed as recombinant proteins. (DOCX) [file pone.0134292.s005.docx]

1. **CLUSTAL W2 multiple sequence alignment of bDBL1 /pCIDR/eDBL5** (eDBL1 was added for comparison)

DBL1: residues 1-437; pCIDR: residues 508-787; DBL5 residues 2025-2321

Amino acids encoded by the cloning sites are in italics, Cysteine residues are in blue

bDBL1 --*DP*MGSSHSTNDAKSPTLSESHKSARNVLENIGIKIYNQEIKKKNPYEQQLKGTLSRAQ 58

eDBL1 *ISEF*MGSSHSTNDAKSPTLSESHKSARNVLENIGIKIYNQEIKKKNPYEQQLKGTLSRAQ 60

eDBL5 *ISE*--------------------------------------------------------- 3

pCIDR *M*--------------------------------------------------------TTF 4

bDBL1 FVDALSSRYGYVRNSDGNSCNLDHLFHTNIKTGYNEGRKPCYGREQNRFDENAEAYCNSD 118

eDBL1 FVDALSSRYGYVRNSDGNSCNLDHLFHTNIKTGYNEGRKPCYGREQNRFDENAEAYCNSD 120

eDBL5 FDNNECKKYGIGSCPEKN------------------------------FHKKLEEWTN-- 31

pCIDR DVDMIYLRRQYMK-------------------------------------NELKSFVEST 27

: : :: : : :

bDBL1 KIRGNENNANGAACAPPRRRHICDQNLEFLDNKNTNTAHDLLGNVLVTAKYEGNYIVNDH 178

eDBL1 KIRGNENNANGAACAPPRRRHICDQNLEFLDNKNTNTAHDLLGNVLVTAKYEGNYIVNDH 180

eDBL5 YVLNNKANKNKAAIVPPRRRQLCLQNLARNLAR-LNKEKSFKEGILISAASEAKMLTEQY 90

pCIDR CLFKGMRNQEWTCQILKNLDVCKLNNFNKVIDIDKHITFKVLLERWLKDFLEG-YKKSKR 86

: . * : :. . :*: : .. :. *. ..

bDBL1 PDKNSNGNKAGICTSLARSFADIGDIVRGRDMFLPNKDDKVQKGLQVVFKKIYKSLTPEA 238

eDBL1 PDKNSNGNKAGICTSLARSFADIGDIVRGRDMFLPNKDDKVQKGLQVVFKKIYKSLTPEA 240

eDBL5 RE-----NPAKALQAIKYSFADIGNIIKG--------DDIIGNVISVQLNKLIN------ 131

pCIDR KINPCTKDKNSCIKLCINKCTCVEEWLNK--------KEKEWGQIKKHFNKQFHG----- 133

: . : : : :. .: :. ::* :

bDBL1 RKHYAHGDGSGNYAKLREDWWTINREQIWKALTCSAPYYADYFRKGSDGTLHFSSHGKCG 298

eDBL1 RKHYAHGDGSGNYAKLREDWWTINREQIWKALTCSAPYYADYFRKGSDGTLHFSSHGKCG 300

eDBL5 ------GNKKINTATL---WWEANKEKIWNAMMC----------------LHWRRKKTAT 166

pCIDR --------EGYDIAFKVKSYFEDNEADVRKSIDN----------------FHVLKN---- 165

: * :: *. .: ::: :* :

bDBL1 HNEGAPPTYLDYVPQFLRWFEEWSEEFCRIKKIKIDKVKKECRDEQNKKYCSGDGHDCTQ 358

eDBL1 HNEGAPPTYLDYVPQFLRWFEEWSEEFCRIKKIKIDKVKKECRDEQNKKYCSGDGHDCTQ 360

eDBL5 SSP--SHDNIDKEDQFLRWFQEWGENFCATRKELYEKLNNECKS---------------- 208

pCIDR ----------KEEYEICNVDDNCRSQNNKKKKDIVTILLKELKD---------------- 199

. :: . :: .: :* : :* :.

bDBL1 TNLAHNQIFVDLDCPRCQDQCIKYNEWIVKKLEEFYKQNLKYSMEIQKWKKTKNNYYDKE 418

eDBL1 TNLAHNQIFVDLDCPRCQDQCIKYNEWIVKKLEEFYKQNLKYSMEIQKWKKTKNNYYDKE 420

eDBL5 --VECNAAKGNVNELKCTKACEEYKSYVLKKKTEYEIQ---------------KDKYDKE 251

pCIDR ---KIVSCKNQHKATKGKECCDKLPKIADGDTSDDEEQ-------------------EDE 237

: . : . * : . . : * :.*

bDBL1 FYENLDKKSYSTIDKFLNLLNNGKHCHDNKDEKNKIDFNKPIKTFSISEYCKTCPLYGVT 478

eDBL1 FYENLDKKSYSTIDKFLNLLNNGKHCHDNKDEKNKIDFNKPIKTFSISEYCKTCPLYGVT 480

eDBL5 FNKALNNK---NALEFLNVQCISEYFSDSKN------WESPYDTFDDDT------LKGTY 296

pCIDR APAPPKPKPPSTPNPCVRKDQSGTHIVSVED--------------------VAEWMQGVT 277

. * . :. . : . :: : *.

bDBL1 CTNRGICIHNS*GAHHHHHH* 497

eDBL1 CTNRGICIHNS-------- 491

eDBL5 DCKKHLVPRGSSA------ 309

pCIDR HDRV-------- *HHHHHH* 287

. :

**identity matrix**

|  | **bDBL1** | **eDBL1** | **eDBL5** | **pCIDR** |
| --- | --- | --- | --- | --- |
| **bDBL1** | 100.00 | 99.59 | 31.07 | 13.24 |
| **eDBL1** | 99.59 | 100.00 | 30.94 | 13.24 |
| **eDBL5** | 31.07 | 30.94 | 100.00 | 9.74 |
| **pCIDR** | 13.24 | 13.24 | 9.74 | 100.00 |

1. **CLUSTAL W2 multiple sequence alignment of eDBL3/pCIDR/eDBL2** (bDBL2 was added for comparison)

pCIDR: residues 508-787; DBL2 residues 821-1242; DBL3 residues1220-1578

amino acids encoded by the cloning sites are in italics, Cysteine residues are in blue

eDBL2 *GS*--SNAVHRSGYNYKGPCTGKNQERFKIGTDWKDANFVSTIHKDFYLPPRREHMCTSNL 58

bDBL2 *DR*WHSNAVHRSGYNYKGPCTGKNQERFKIGTDWKDANFVSTIHKDFYLPPRREHMCTSNL 60

eDBL3 *GS*TSACDIVKNLLDGKGETSSIYGCNIKSDVNWECEKNIDPKYTGACMPPRRQKLCVSSL 60

pCIDR -------*M*TTFDVDMIYLRRQYMKNELKSFVESTCLFKGMRNQEWTCQILKNLDVCKLNN 53

: : .:* .: :. .:* .

eDBL2 EK---LDVQRVIGYSNVNDAFLGDVLVAAKFEGEN--IKERLKKYPADQTICRSMRYSFA 113

bDBL2 EK---LDVQRVIGYSNVNDAFLGDVLVAAKFEGEN--IKERLKKYPADQTICRSMRYSFA 115

eDBL3 TKQRNIKEKEDIRTEFIKSAAIETHFAWYKYKKDNDKAERELNSGIIPEGFIRQMYYTFG 120

pCIDR FN-KVIDIDKHITFKVLLERWLKDFLEGYKKSKRK------INPCTKDKNSCIKLCINKC 106

: :. .. * . : . : : * . : :: : .: .

eDBL2 DIGDIIRGKDLWDKDNGAKDMEKHLVSIFKKIKEKHYGIKDNPKYEKGGEPFTKLRSDWW 173

bDBL2 DIGDIIRGKDLWDKDNGAKDMEKHLVSIFKKIKEKHYGIKDNPKYEKGGEPFTKLRSDWW 175

eDBL3 DYRDIFFGTDISKHSHISR-----VSSSVKDILKEESKERKNP-------------EEWW 162

pCIDR TCVEEWLNKKEKEWGQIKKHFNKQFHGEGYDIAFKVKS--------------------YF 146

: ... . .: : . . .* : ::

eDBL2 SANRDQIWKAMQCQTLGPRVINTCDKEPTPIDDYI--PQRLRWMTEWAEWFCKAQNKYYG 231

bDBL2 SANRDQIWKAMQCQTLGPRVINTCDKEPTPIDDYI--PQRLRWMTEWAEWFCKAQNKYYG 233

eDBL3 NEHGKEIWKAMLCALPGTGNLQNNPDYNNPPEDVAKKPQFLRWFTEWGEDFCRTRGVKIK 222

pCIDR EDNEADVRKSIDN-------------------------------------FHVLKNKEEY 169

. : :: *:: * :.

eDBL2 DLVGACRKCQEKKTECTQGTSQCDTCKEKCTKYNDFIKKWQPQWKQMEQKYKDLYGKAKI 291

bDBL2 DLVGACRKCQEKKTECTQGTSQCDTCKEKCTKYNDFIKKWQPQWKQMEQKYKDLYGKAKI 293

eDBL3 ELEKGCAGYECDVTDETK----KEACKKACEKYQTWLKDWKTQYEKRSQKFTRDKGKPEY 278

pCIDR EICNVDDNCRS------------QNNKKKKDIVTILLKELKDKIVSCKNQHKATKGK--- 214

:: . : *: :*. : : . .::.. **

eDBL2 AANGDETVVPNGHTTDKDQQVVNFLKLLIPRSDKSGSKSGNTPYSTAGGYIHETADVNNC 351

bDBL2 AANGDETVVPNGHTTDKDQQVVNFLKLLIPRSDKSGSKSGNTPYSTAGGYIHETADVNNC 353

eDBL3 EVGPDVASSQN---------AYKYLSKKLKSICQNGATTEKCDYNCMENAARQ-PQTSAS 328

pCIDR ----------------------ECCDKLPKIADGDTSDDEEQEDEAPAPPKPKPPSTPNP 252

: . . : : . : ...

eDBL2 NTQKVFCNTASKDNYAFQLTPKGYEQECMCEKRPKAPQKKPEVPKVKPPTSACDIVKNLL 411

bDBL2 NTQKVFCNTASKDNYAFQLTPKGYEQECMCEKRPKAPQKKPEVPKVKPPTSACDIVKNLL 413

eDBL3 SDQQENSATQKDLPEAFDCPPKEIGDRCNCPK---------------------------- 360

pCIDR CVRKDQSGTHIVSVEDVAEWMQGVTHD--------------------------------- 279

:: . * . : .

eDBL2 DGKGETSSIYG*LEHHHHHH* 430

bDBL2 DGKGETSSIYG*LEHHHHHH* 432

eDBL3 ----------LLEHHHHHH 369

pCIDR -----------RV*HHHHHH* 287

******

**identity matrix**

|  | **eDBL2** | **bDBL2** | **eDBL3** | **pCIDR** |
| --- | --- | --- | --- | --- |
| **eDBL2** | 100.00 | 99.53 | 25.56 | 14.39 |
| **bDBL2** | 99.53 | 100.00 | 25.14 | 14.39 |
| **eDBL3** | 25.56 | 25.14 | 100.00 | 14.59 |
| **pCIDR** | 14.39 | 14.39 | 14.59 | 100.00 |

1. **CLUSTAL W2 multiple sequence alignment of eDBL2/eDBL3**

DBL2: residues 821-1242; DBL3: residues 1220-157 (bDBL2 was added for comparison)

Amino acids encoded by the cloning sites are in italics, Cysteine residues are in blue

eDBL2 *GS-*-SNAVHRSGYNYKGPCTGKNQERFKIGTDWKDANFVSTIHKDFYLPPRREHMCTSNL 58

bDBL2 *DR*WHSNAVHRSGYNYKGPCTGKNQERFKIGTDWKDANFVSTIHKDFYLPPRREHMCTSNL 60

eDBL3 *GS*TSACDIVKNLLDGKGETSSIYGCNIKSDVNWECEKNIDPKYTGACMPPRRQKLCVSSL 60

: : :. : ** :. .:* ..:*: : :.. :.. :****:::*.*.*

eDBL2 EK---LDVQRVIGYSNVNDAFLGDVLVAAKFEGEN--IKERLKKYPADQTICRSMRYSFA 113

bDBL2 EK---LDVQRVIGYSNVNDAFLGDVLVAAKFEGEN--IKERLKKYPADQTICRSMRYSFA 115

eDBL3 TKQRNIKEKEDIRTEFIKSAAIETHFAWYKYKKDNDKAERELNSGIIPEGFIRQMYYTFG 120

* :. :. * . ::.* : :. *:: :* :..*:. : : *.* *:*.

eDBL2 DIGDIIRGKDLWDKDNGAKDMEKHLVSIFKKIKEKHYGIKDNPKYEKGGEPFTKLRSDWW 173

bDBL2 DIGDIIRGKDLWDKDNGAKDMEKHLVSIFKKIKEKHYGIKDNPKYEKGGEPFTKLRSDWW 175

eDBL3 DYRDIFFGTDISKHSHISR-----VSSSVKDILKEESKERKNP-------------EEWW 162

* **: *.*: .:.: :: : * .*.* ::. :.** .:**

eDBL2 SANRDQIWKAMQCQTLGPRVINTCDKEPTPIDDYI--PQRLRWMTEWAEWFCKAQNKYYG 231

bDBL2 SANRDQIWKAMQCQTLGPRVINTCDKEPTPIDDYI--PQRLRWMTEWAEWFCKAQNKYYG 233

eDBL3 NEHGKEIWKAMLCALPGTGNLQNNPDYNNPPEDVAKKPQFLRWFTEWGEDFCRTRGVKIK 222

. : .:***** * *. ::. . .* :* ** ***:***.* **:::.

eDBL2 DLVGACRKCQEKKTECTQGTSQCDTCKEKCTKYNDFIKKWQPQWKQMEQKYKDLYGKAKI 291

bDBL2 DLVGACRKCQEKKTECTQGTSQCDTCKEKCTKYNDFIKKWQPQWKQMEQKYKDLYGKAKI 293

eDBL3 ELEKGCAGYECDVTDETK----KEACKKACEKYQTWLKDWKTQYEKRSQKFTRDKGKPEY 278

:* .* : . *: *: ::**: * **: ::*.*:.*::: .**:. **.:

eDBL2 AANGDETVVPNGHTTDKDQQVVNFLKLLIPRSDKSGSKSGNTPYSTAGGYIHETADVNNC 351

bDBL2 AANGDETVVPNGHTTDKDQQVVNFLKLLIPRSDKSGSKSGNTPYSTAGGYIHETADVNNC 353

eDBL3 EVGPDVASSQN---------AYKYLSKKLKSICQNGATTEKCDYNCMENAARQ-PQTSAS 328

.. * : * . ::*. : :.*:.: : *. . :: .:.. .

eDBL2 NTQKVFCNTASKDNYAFQLTPKGYEQECMCEKRPKAPQKKPEVPKVKPPTSACDIVKNLL 411

bDBL2 NTQKVFCNTASKDNYAFQLTPKGYEQECMCEKRPKAPQKKPEVPKVKPPTSACDIVKNLL 413

eDBL3 SDQQENSATQKDLPEAFDCPPKEIGDRCNCPK---------------------------- 360

. *: . * .. **: .** :.* * *

eDBL2 DGKGETSSIYG*LEHHHHHH* 430

bDBL2 DGKGETSSIYG*LEHHHHHH* 432

eDBL3 ----------L*LEHHHHHH* 369

********

| **identity matrix** |  |  |  |
| --- | --- | --- | --- |
|  | **eDBL2** | **bDBL2** | **eDBL3** |
| **eDBL2** | 100.00 | 99.53 | 25.56 |
| **bDBL2** | 99.53 | 100.00 | 25.14 |
| **eDBL3** | 25.56 | 25.14 | 100.00 |

1. **CLUSTAL W2 multiple sequence alignment of eDBL1/eDBL2 /bDBL2** (bDBL1 was added for comparison) DBL1: residues 1-437 ; DBL2: residues 821-1242

Amino acids encoded by the cloning sites are in italics, Cysteine residues are in blue

eDBL2 ------------------------------------------------------------

bDBL2 ------------------------------------------------------------

bDBL1 --*DP*MGSSHSTNDAKSPTLSESHKSARNVLENIGIKIYNQEIKKKNPYEQQLKGTLSRAQ 58

eDBL1 *ISEF*MGSSHSTNDAKSPTLSESHKSARNVLENIGIKIYNQEIKKKNPYEQQLKGTLSRAQ 60

eDBL2 -----------------------*GS*SNAVHRSGYN-YKGPCTGKNQERFKIGTDWKDAN- 35

bDBL2 ---------------------*DR*WHSNAVHRSGYN-YKGPCTGKNQERFKIGTDWKDAN- 37

bDBL1 FVDALSSRYGYVRNSDGNSCNLDHLFHTNIKTGYNEGRKPCYGREQNRFDENAEAYCNSD 118

eDBL1 FVDALSSRYGYVRNSDGNSCNLDHLFHTNIKTGYNEGRKPCYGREQNRFDENAEAYCNSD 120

:: ::*** : ** *::*:**. .:: .

eDBL2 ----FVSTIHKDFYLPPRREHMCTSNLEKLDVQRVIGYSNVNDAFLGDVLVAAKFEGENI 91

bDBL2 ----FVSTIHKDFYLPPRREHMCTSNLEKLDVQRVIGYSNVNDAFLGDVLVAAKFEGENI 93

bDBL1 KIRGNENNANGAACAPPRRRHICDQNLEFLDNK----NTNTAHDLLGNVLVTAKYEGNYI 174

eDBL1 KIRGNENNANGAACAPPRRRHICDQNLEFLDNK----NTNTAHDLLGNVLVTAKYEGNYI 176

.. : ****.*:* .*** ** : :*. . :**:***:**:**: *

eDBL2 KERLKKYPADQT---ICRSMRYSFADIGDIIRGKDLWDKDNGAKDMEKHLVSIFKKIKEK 148

bDBL2 KERLKKYPADQT---ICRSMRYSFADIGDIIRGKDLWDKDNGAKDMEKHLVSIFKKIKEK 150

bDBL1 VNDHPDKNSNGNKAGICTSLARSFADIGDIVRGRDMFLPNKDDKVQKGLQVVFKKIYKSL 234

eDBL1 VNDHPDKNSNGNKAGICTSLARSFADIGDIVRGRDMFLPNKDDKVQKGLQVVFKKIYKSL 236

: . :: . ** *: ********:**:*:: ::. * : * : * *.

eDBL2 HYGIKDNPKYEKGGEPFTKLRSDWWSANRDQIWKAMQCQT--------------LGPRVI 194

bDBL2 HYGIKDNPKYEKGGEPFTKLRSDWWSANRDQIWKAMQCQT--------------LGPRVI 196

bDBL1 TPEARKHYAHGDGSGNYAKLREDWWTINREQIWKALTCSAPYYADYFRKGSDGTLHFSSH 294

eDBL1 TPEARKHYAHGDGSGNYAKLREDWWTINREQIWKALTCSAPYYADYFRKGSDGTLHFSSH 296

:.: : .*. ::***.***: **:*****: *.: *

eDBL2 NTCDK---EPTPIDDYIPQRLRWMTEWAEWFCKAQNKYYGDLVGACRKCQEKKTECTQGT 251

bDBL2 NTCDK---EPTPIDDYIPQRLRWMTEWAEWFCKAQNKYYGDLVGACRKCQEKKTECTQGT 253

bDBL1 GKCGHNEGAPPTYLDYVPQFLRWFEEWSEEFCRIKKIKIDKVKKECRDEQNKKYCSGDG- 353

eDBL1 GKCGHNEGAPPTYLDYVPQFLRWFEEWSEEFCRIKKIKIDKVKKECRDEQNKKYCSGDG- 355

..*.: *.. **:** ***: **:* **: :: ..: **. *:** . :*

eDBL2 SQCDTCKEKCTKYNDFIKKWQPQWKQMEQKYKDLYGKAKIAANGDETVVPNGHTTDKDQQ 311

bDBL2 SQCDTCKEKCTKYNDFIKKWQPQWKQMEQKYKDLYGKAKIAANGDETVVPNGHTTDKDQQ 313

bDBL1 --HDCTQTNLAHNQIFVDLDCPRCQDQCIKYNEWIVKKLEEFYKQNLKYSMEIQKWKKTK 411

eDBL1 --HDCTQTNLAHNQIFVDLDCPRCQDQCIKYNEWIVKKLEEFYKQNLKYSMEIQKWKKTK 413

* : : :: : *:. *: :: **:: * :: . . *. :

eDBL2 VVNFLKLLIPRSDKSGSKSGNTPYSTAGGYIHETADVNNCNTQKVFCNTASKDNYAFQLT 371

bDBL2 VVNFLKLLIPRSDKSGSKSGNTPYSTAGGYIHETADVNNCNTQKVFCNTASKDNYAFQLT 373

bDBL1 NNYYDKEFYENLDK-------KSYSTIDKFLNLLNNGKHCHD-----NKDEKNKIDFNKP 459

eDBL1 NNYYDKEFYENLDK-------KSYSTIDKFLNLLNNGKHCHD-----NKDEKNKIDFNKP 461

: * : . ** ..*** . ::: : ::*: *. .*:: *: .

eDBL2 PKGYEQECMCEKRPKAPQKKPEVPKVKPPTSACDIVKNLLDGKGETSSIYG*LEHHHHHH* 430

bDBL2 PKGYEQECMCEKRPKAPQKKPEVPKVKPPTSACDIVKNLLDGKGETSSIYG*LEHHHHHH* 432

bDBL1 IKTFSISEYCKT--------------------CPLYGVTCTNRGICIHNS*GAHHHHHH*- 497

eDBL1 IKTFSISEYCKT--------------------CPLYGVTCTNRGICIHNS--------- 491

* :. . *:. * : .:*

| **identity matrix** |  |  |  |  |
| --- | --- | --- | --- | --- |
|  | **eDBL2** | **bDBL2** | **bDBL1** | **eDBL1** |
| **eDBL2** | 100.00 | 99.53 | 28.72 | 27.75 |
| **bDBL2** | 99.53 | 100.00 | 28.57 | 27.60 |
| **bDBL1** | 28.72 | 28.57 | 100.00 | 99.59 |
| **eDBL1** | 27.75 | 27.60 | 99.59 | 100.00 |

1. **CLUSTAL W2 multiple sequence alignment of individualPfEMP1 VarO domains expressed as recombinant protein** (excluding amino acids encoded by the cloning sites and the histidinine tag) (see Table 1) Cysteine residues are in blue.

DBL0 ------------------------------------------------------------

DBL1 MGSSHSTNDAKSPTLSESHKSARNVLENIGIKIYNQEIKKKNPYEQQLKGTLSRAQFVDA 60

bCIDR --------------SMEIQKWKKTKNNYYDKEFYENLDKKSYSTIDKFLNLLNNGKHCHD 46

pCIDR ------------------------------------------------------------

DBL2 ------------------------------------------------------------

DBL4 --------------------------------------------------------VNTY 4

DBL5 ------------------------------------------------------------

DBL3 ------------------------------------------------------------

DBL0 ----------------------------------KPCYGREQNRFDENAEAYCNSDKIRG 26

DBL1 LSSRYGYVRNSDGNSCNLDHLFHTNIKTGYNEGRKPCYGREQNRFDENAEAYCNSDKIRG 120

bCIDR NKDEKNKIDFNKPIKTFSISEYCKTCPLYGVTCTNRGICIHNSNNKNKGENDLNKINIKD 106

pCIDR ------------------------------------------------------------

DBL2 ---------------------HSNAVHRSGYNYKGPCTGKNQERFKIG-----TDWKDAN 34

DBL4 NDNCKNAKREDYANQNGETCKFKEVSWSSIGIINNENEATGRDRFKIG-----EVWECNK 59

DBL5 --------------------DNNECKKYGIGSCPEKNFHKKLEEWTNY---------VLN 31

DBL3 ------------------TSACDIVKNLLDGKGETSSIYGCNIKSDVN-------WECEK 35

DBL0 NENNSNGTACAPPRRRHICDQNLEFLDNK----NTNTTHDLLGNVLVTAKYEGNYIVNDH 82

DBL1 NENNANGAACAPPRRRHICDQNLEFLDNK----NTNTAHDLLGNVLVTAKYEGNYIVNDH 176

bCIDR KSPTTFDVDMIYLRRQYMKNELKSFVEST----CLFKGMRNQEWTCQILKNLDVCKLNNF 162

pCIDR ---TTFDVDMIYLRRQYMKNELKSFVEST----CLFKGMRNQEWTCQILKNLDVCKLNNF 53

FVSTIHKDFYLPPRREHMCTSNLEKLDVQRVIGYSNVNDAFLGDVLVAAKFEGENIKERL 94

DBL4 ETTDGKNKVCVPPRRKDMCLKKLQDIRVD----DISDSSTLLKEIQEVAKNEGNDIIRNL 115

DBL5 NKANKNKAAIVPPRRRQLCLQNLARNLAR-----LNKEKSFKEGILISAASEAKMLTEQY 86

DBL3 NIDPKYTGACMPPRRQKLCVSSLTKQRNIK--EKEDIRTEFIKSAAIETHFAWYKYKKDN 93

DBL0 PDKNSNGNK----SGICTSLARSFADIGDIVRGRDMFLPNKDDKVQKGLQVVFKKIYKSL 138

DBL1 PDKNSNGNK----AGICTSLARSFADIGDIVRGRDMFLPNKDDKVQKGLQVVFKKIYKSL 232

bCIDR NKVIDIDKH----ITFKVLLERWLKDFLEGYKKSKRKINPCTKDKNSCIKLCINKCTCVE 218

pCIDR NKVIDIDKH----ITFKVLLERWLKDFLEGYKKSKRKINPCTKDKNSCIKLCINKCTCVE 109

DBL2 KKYPADQT-------ICRSMRYSFADIGDIIRGKDLWDKDNGAKDMEKHLVSIFKKIKEK 147

LP-KYPCNE----DVICKYMKYSFADLGDIVRGTDKYKDVIGSISSGNNAEQIEENLKTI 170

DBL5 RE-----NP----AKALQAIKYSFADIGNIIKGDDIIGN------------VISVQLNKL 125

DBL3 DKAERELNSGIIPEGFIRQMYYTFGDYRDIFFGTDISKHS--------HISRVSSSVKDI 145

DBL0 TPEARKHYA-HGDGSGNYSKLREDWWTINREQIWKALTCSAP-----------------Y 180

DBL1 TPEARKHYA-HGDGSGNYAKLREDWWTINREQIWKALTCSAP-----------------Y 274

bCIDR EWLNKKEKE-WGQIKKHFN---KQFHGEGYDIAFKVKSYFED-----------------N 257

pCIDR EWLNKKEKE-WGQIKKHFN---KQFHGEGYDIAFKVKSYFED-----------------N 148

DBL2 HYGIKDNPK-YEKGGEPFTKLRSDWWSANRDQIWKAMQCQTLGPRVINTCDKEPTPIDDY 206

DBL4 FENIQKTDENFQKKYTNLELFRSAWWDANRKDIWKAMTCNAP-----------------D 213

DBL5 INGNKKINT------------ATLWWEANKEKIWNAMMCLHWR----------------- 156

DBL3 LKEESKERK-----------NPEEWWNEHGKEIWKAMLCALPG----------------- 177

DBL0 YADYFRKGSDGTLHFS--SHGKCGHNEGAPPTYLDYVPQFLR-WFEEWSEEFCRIKKIKI 237

DBL1 YADYFRKGSDGTLHFS--SHGKCGHNEGAPPTYLDYVPQFLR-WFEEWSEEFCRIKKIKI 331

bCIDR EADVRKSIDNFHVLKNKEEYEICNVDDNCRSQNNKKKKDIVTILLKELKDKIVSCKNQHK 317

pCIDR EADVRKSIDNFHVLKNKEEYEICNVDDNCRSQNNKKKKDIVTILLKELKDKIVSCKNQHK 208

DBL2 IPQRLRWMTEWAEWFCKAQNKYYGDLVGACRKCQEKKTECTQGTSQCDTCKEKCTKYNDF 266

DBL4 EAKIYITKEGGYISPITWTKNHCGHNDDPPD--YDYIPQPLR-WISEWSESYCLAQKDFL 270

DBL5 --------------------RKKTATSSPSHDNIDKEDQFLR-WFQEWGENFCATRKELY 195

-----------------TGNLQNNPDYNNPPEDVAKKPQFLR-WFTEWGEDFCRTRGVKI 219

DBL0 DKVKKECRDEQNKKYCSGDGHDCTQTNLSHNQIFVDLDCPRCQDQCIKYNEWIVKKLEEF 297

DBL1 DKVKKECRDEQNKKYCSGDGHDCTQTNLAHNQIFVDLDCPRCQDQCIKYNEWIVKKLEEF 391

bCIDR ATKGKECCDKLPKIADGDTSDDEEQEDEAP--APPKPKPPSTPNPCVRKDQSGTHIVSVE 375

pCIDR ATKGKECCDKLPKIADGDTSDDEEQEDEAP--APPKPKPPSTPNPCVRKDQSGTHIVSVE 266

IKKWQPQWKQMEQKYKDLYGKAKIAANGDE-TVVPNGHTTDKDQQVVNFLKLLIPRSDKS 325

ETMKN---CENCKKKNDNTDCEQTKYGACRDCKKKCEEYKKFVDKWKAQFETQNKAYKEI 327

DBL5 EKLNNECKSVECNAAKGNVNELKCTKACEEYKSYVLKKKTEYEIQKDKYDKEFNKALNNK 255

KELEKGCAGYECDVT-DETKKEACKKACEKYQTWLKDWKTQYEKRSQKFTRDKGKPEYEV 278

DBL0 YKQNLKYSMEI------------------------------------------------- 308

DBL1 YKQNLKYSMEIQKWKKTKNNYYDKEFYENLDKKSYSTIDKFLN-LLNNGKHCHDNKDEKN 450

bCIDR DVAEWMQGVTHDRVKDVDGLKADASKGTYSRMGSPSAFNDICN-IEIKHSNAVHRSGYNY 434

pCIDR DVAEWMQGVTHDRV---------------------------------------------- 280

DBL2 GSKSGNTPYSTAGGYIHETADVNNCNTQKVFCNTASKDNYAFQ-LTPKGYEQECMCEKRP 384

DBL4 YKNAATSSGRHSNGIDEDIKKFVEKLEQNCQKNSVDTADKYLE-GGSVCRRFKFVKTDTH 386

DBL5 NALEFLNVQCISEYFSDS---------KNWESPYDTFDDDTLK-GTYDCKKH-------- 297

DBL3 G-PDVASSQNAYKYLSKKLKSICQNGATTEKCDYNCMENAARQPQTSASSDQQENSATQK 337

DBL0 --------------------------------------

DBL1 KIDFNKPIKTFSISEYCKTCPLYGVTCTNRGICIHNS- 487

bCIDR KGP----------------------------------- 437

pCIDR --------------------------------------

KAPQKKP-EVPKVKPPTSACDIVKNLLDGKGETSSIYG 421

EKNYAFHNTPLSYKEHCECAK----------------- 407

DBL5 --------------------------------------

DLPEAFDCPPKEIGDRCNCPKL---------------- 359

**identity matrix**

|  | **DBL0** | **DBL1** | **bCIDR** | **pCIDR** | **DBL2** | **DBL4** | **DBL5** | **DBL3** |
| --- | --- | --- | --- | --- | --- | --- | --- | --- |
| **DBL0** | 100.00 | 98.05 | 9.57 | 9.49 | 25.08 | 26.60 | 24.70 | 21.59 |
| **DBL1** | 98.05 | 100.00 | 9.68 | 9.75 | 20.96 | 23.02 | 23.65 | 18.29 |
| **bCIDR** | 9.57 | 9.68 | 100.00 | 100.00 | 7.73 | 7.57 | 8.53 | 8.84 |
| **pCIDR** | 9.49 | 9.75 | 100.00 | 100.00 | 7.58 | 7.33 | 9.52 | 8.26 |
| **DBL2** | 25.08 | 20.96 | 7.73 | 7.58 | 100.00 | 16.22 | 17.81 | 14.45 |
| **DBL4** | 26.60 | 23.02 | 7.57 | 7.33 | 16.22 | 100.00 | 21.31 | 19.48 |
| **DBL5** | 24.70 | 23.65 | 8.53 | 9.52 | 17.81 | 21.31 | 100.00 | 22.71 |
| **DBL3** | 21.59 | 18.29 | 8.84 | 8.26 | 14.45 | 19.48 | 22.71 | 100.00 |
